# Incarceration history and risk of HIV and hepatitis C virus acquisition among people who inject drugs: a systematic review and meta-analysis

**DOI:** 10.1016/S1473-3099(18)30469-9

**Published:** 2018-12

**Authors:** Jack Stone, Hannah Fraser, Aaron G Lim, Josephine G Walker, Zoe Ward, Louis MacGregor, Adam Trickey, Sam Abbott, Steffanie A Strathdee, Daniela Abramovitz, Lisa Maher, Jenny Iversen, Julie Bruneau, Geng Zang, Richard S Garfein, Yung-Fen Yen, Tasnim Azim, Shruti H Mehta, Michael-John Milloy, Margaret E Hellard, Rachel Sacks-Davis, Paul M Dietze, Campbell Aitken, Malvina Aladashvili, Tengiz Tsertsvadze, Viktor Mravčík, Michel Alary, Elise Roy, Pavlo Smyrnov, Yana Sazonova, April M Young, Jennifer R Havens, Vivian D Hope, Monica Desai, Ellen Heinsbroek, Sharon J Hutchinson, Norah E Palmateer, Andrew McAuley, Lucy Platt, Natasha K Martin, Frederick L Altice, Matthew Hickman, Peter Vickerman

**Affiliations:** a Population Health Sciences, Bristol Medical School, University of Bristol, Bristol, UK; b Division of Infectious Diseases and Global Public Health, University of California San Diego, La Jolla, CA, USA; c Kirby Institute for Infection and Immunity, Faculty of Medicine, University of New South Wales, Sydney, NSW, Australia; d Department of Family Medicine, Université de Montréal, Montréal, QC, Canada; e Centre hospitalier de l'Université de Montreal, Montréal, QC, Canada; f Section of Infectious Diseases, Taipei City Hospital, Taipei City Government, Taipei, Taiwan; g James P Grant School of Public Health, BRAC University, Dhaka, Bangladesh; h Department of Epidemiology, Johns Hopkins Bloomberg School of Public Health, Baltimore, MD, USA; i BC Centre for Excellence in HIV/AIDS and Division of AIDS, Faculty of Medicine, University of British Columbia, Vancouver, BC, Canada; j Burnet Institute, Melbourne, VIC, Australia; k Department of Epidemiology and Preventive Medicine, Monash University, Melbourne, VIC, Australia; l Department of Medicine, University of Melbourne, Melbourne, VIC, Australia; m Infectious Diseases, AIDS and Clinical Immunology Research Center, Tbilisi, Georgia; n Faculty of Medicine, Tbilisi State University, Tbilisi, Georgia; o National Monitoring Centre for Drugs and Addiction, Prague, Czech Republic; p Department of Addictology, The First Medical Faculty, Charles University and General University Hospital in Prague, Prague, Czech Republic; q National Institute of Mental Health, Klecany, Czech Republic; r University Hospital Centre of Québec Research Centre—Laval University, QC, Canada; s National Institute of Public Health of Québec, QC, Canada; t Faculty of Medicine and Health Sciences, University of Sherbrooke, Longueuil, QC, Canada; u International Charitable Foundation Alliance for Public Health, Kiev, Ukraine; v Department of Epidemiology, University of Kentucky College of Public Health, KY, USA; w Center on Drug and Alcohol Research, University of Kentucky, KY, USA; x Public Health Institute, Liverpool John Moores University, Liverpool, UK; y National Infection Service, Public Health England, London, UK; z School of Health and Life Sciences, Glasgow Caledonian University, Glasgow, UK; aa Health Protection Scotland, National Health Service National Services Scotland, Glasgow, UK; ab Faculty of Public Health and Policy, London School of Hygiene and Tropical Medicine, London, UK; ac Yale School of Medicine, Department of Medicine, Section of Infectious Diseases, AIDS Program, New Haven, CT, USA

## Abstract

**Background:**

People who inject drugs (PWID) experience a high prevalence of incarceration and might be at high risk of HIV and hepatitis C virus (HCV) infection during or after incarceration. We aimed to assess whether incarceration history elevates HIV or HCV acquisition risk among PWID.

**Methods:**

In this systematic review and meta-analysis, we searched MEDLINE, Embase, and PsycINFO databases for studies in any language published from Jan 1, 2000 until June 13, 2017 assessing HIV or HCV incidence among PWID. We included studies that measured HIV or HCV incidence among community-recruited PWID. We included only studies reporting original results and excluded studies that evaluated incident infections by self-report. We contacted authors of cohort studies that met the inclusion or exclusion criteria, but that did not report on the outcomes of interest, to request data. We extracted and pooled data from the included studies using random-effects meta-analyses to quantify the associations between recent (past 3, 6, or 12 months or since last follow-up) or past incarceration and HIV or HCV acquisition (primary infection or reinfection) risk among PWID. We assessed the risk of bias of included studies using the Newcastle-Ottawa Scale. Between-study heterogeneity was evaluated using the *I*^2^ statistic and the P-value for heterogeneity.

**Findings:**

We included published results from 20 studies and unpublished results from 21 studies. These studies originated from Australasia, western and eastern Europe, North and Latin America, and east and southeast Asia. Recent incarceration was associated with an 81% (relative risk [RR] 1·81, 95% CI 1·40–2·34) increase in HIV acquisition risk, with moderate heterogeneity between studies (*I*^2^=63·5%; p=0·001), and a 62% (RR 1·62, 95% CI 1·28–2·05) increase in HCV acquisition risk, also with moderate heterogeneity between studies (*I*^2^=57·3%; p=0·002). Past incarceration was associated with a 25% increase in HIV (RR 1·25, 95% CI 0·94–1·65) and a 21% increase in HCV (1·21, 1·02–1·43) acquisition risk.

**Interpretation:**

Incarceration is associated with substantial short-term increases in HIV and HCV acquisition risk among PWID and could be a significant driver of HCV and HIV transmission among PWID. These findings support the need for developing novel interventions to minimise the risk of HCV and HIV acquisition, including addressing structural risks associated with drug laws and excessive incarceration of PWID.

**Funding:**

Engineering and Physical Sciences Research Council, National Institute for Health Research, National Institutes of Health.

## Introduction

Hepatitis C virus (HCV) and HIV are leading causes of morbidity and mortality and continue to represent major global public health concerns.^1^, ^2^ Injecting drug use is associated with two-fifths of the global HCV disease burden,^3^ while outside sub-Saharan Africa, an estimated one-fifth of new HIV infections occur among people who inject drugs (PWID).^4^

PWID have a high prevalence of incarceration (58% have ever been incarcerated^5^), with a history of incarceration frequently being associated with prevalent HIV and HCV infection.^6^ The risk of relapse to illicit drug use is high in the period immediately following release from prison,^7^, ^8^ and so individuals are at an increased risk of multiple adverse outcomes during this period—in particular drug-related deaths,^9^ but also increased injecting risk behaviours and homelessness,^10^, ^11^, ^12^, ^13^ and reduced access to interventions such as opioid substitution therapy and HIV antiretroviral therapy.^12^, ^14^

Several recent modelling analyses have suggested that incarceration of PWID could be an important contributor to HIV and HCV transmission among PWID, largely owing to the high prevalence of incarceration among this group and an elevated transmission risk following release.^11^, ^15^, ^16^, ^17^ Furthermore, these studies suggest that the period following release could be a key prevention target for reducing the transmission of HIV and HCV among PWID. However, the magnitude and mechanism of this elevated risk following incarceration is not well understood, and there is scarce empirical evidence to support existing modelling, inform policy change, or aid in the development of interventions that target this period of risk.

**Research in context**

**Evidence before the study**

We searched PubMed up to Jan 17, 2018, for “HIV OR hepatitis C OR HCV” AND “incarceration” AND “inject drugs, injecting drug, substance abuse, intravenous/epidemiology [MeSH]” OR “substance-related disorders/epidemiology [MeSH]”, with no restrictions on language or date. The findings of identified studies suggest that previous incarceration is negatively associated with injecting cessation (two studies) and being on opioid substitution therapy (three studies), but is positively associated with prevalent HIV and hepatitis C virus (HCV) infection (17 studies), police harassment (one study), relapse to injecting drug use (one study), unstable housing or homelessness (three studies), overdose (four studies), mortality (one study), and several high-risk, drug-using behaviours such as receptive syringe sharing, public injecting, and cocaine injecting (16 studies). We identified a systematic review on the associations between criminalisation of drug use and HIV prevention and treatment-related outcomes among people who inject drugs (PWID), which included only one study measuring the association between incarceration history and HIV acquisition risk among PWID. In addition to the study identified in that review, our search found five further studies presenting the association between incarceration (either recent or past) and HIV acquisition risk, as well as three studies presenting the association between incarceration (either recent or past) and HCV acquisition risk; all of these studies were also identified in our systematic review. The search also identified three mathematical modelling studies (two done by our team) that suggest that incarceration could substantially contribute to HIV and HCV transmission among PWID, and that scaling up prison-based opioid substitution therapy with retention following release could be an effective prevention strategy.

**Added value of this study**

To our knowledge, this is the first systematic review and meta-analysis of the effect of incarceration on HIV or HCV acquisition risk among community-based PWID. Our study also builds on previously published evidence through collating and synthesising unpublished estimates by contacting authors of all identified studies of PWID that have a measure of HIV or HCV incidence. This approach resulted in 28 additional estimates being included in our meta-analysis, doubling the overall number of studies. We found that recent (past 3, 6, or 12 months or since last follow-up) incarceration was associated with an increased risk of both HIV and HCV acquisition among community-based PWID. These associations persisted when only adjusted estimates were pooled, as well as in numerous sensitivity analyses. Past incarceration was only weakly associated with elevated HIV or HCV acquisition risk, with there being no association when only adjusted estimates are pooled. The association between recent incarceration and HCV acquisition risk was greater in studies with higher prevalences of homelessness and in countries with higher prevalences of incarceration and reduced in studies adjusting for homelessness.

**Implications of all the available evidence**

Evidence suggests that incarceration is an important enhancer of HIV and HCV acquisition risk among PWID globally and probably a significant driver of HIV and HCV transmission among PWID in many settings because of the high prevalence of incarceration among this group. Research is now required to better understand how incarceration elevates HIV and HCV acquisition risk. This research will be useful for guiding the development of interventions to mitigate this risk, with our findings suggesting that interventions are needed to address the social vulnerabilities experienced by PWID when they are released from prison. Our study strengthens the evidence of the harms caused by the criminalisation of drug users, which results in a high prevalence of incarceration among people who use drugs, and provides further evidence to support minimising the use of criminal sanctions to manage drug-use disorders.

To improve the evidence base, we did a systematic review and meta-analysis to quantify the association between incarceration history, either past or recent, and HIV or HCV acquisition risk among PWID.

## Methods

### Search strategy and selection criteria

We did searches of MEDLINE, Embase, and PsycINFO databases without language restrictions. Search terms included those related to HIV infection or transmission, HCV infection or transmission, injecting drug use, and study designs that could be used to evaluate HIV or HCV incidence (a full list of search terms is provided in the appendix). We initially searched up to June 6, 2016, but subsequently updated our search to include studies published up to June 13, 2017. Studies, including those from conference abstracts, were limited to those published since 2000. Reference lists of systematic reviews were hand-searched for additional relevant papers or reports.

An Endnote library was created to catalogue the search results, with removal of duplicates. Titles and abstracts were screened for potential relevance, with full texts obtained and further screened for those deemed relevant. This screening was done by one author (JS), with a subset of references (10%) also screened by other authors (SA, HF, AGL, LMac, AT, JGW, and ZW). No discrepancies were found between the two lists of accepted references and so no further double screening was done. We read non-English full text papers using Google Translate.

The key outcomes of interest were the association between recent (past 3, 6, or 12 months or since last follow-up) or past incarceration and HIV or HCV incidence (either primary infection or reinfection) among PWID—that is, the difference in the risk of acquiring HIV or HCV infection among recently incarcerated PWID compared with PWID without recent incarceration, or among PWID who have previously been incarcerated compared with PWID who have never been incarcerated. Throughout this paper, we use the term incarceration to refer to the detention of people in prisons, jails (in the US and other settings, jails are typically used for short-term detention, either for those awaiting trial or with short sentences, whereas prisons are used to detain those with longer sentences), or other closed settings and use the term prison to refer to any such setting where someone might be detained.

We included studies that measured HIV or HCV incidence among community-recruited samples of PWID, either current or former injectors. We included studies that measured incidence by repeated testing or that used biological markers of recent HIV or HCV infection to estimate incidence. Studies that evaluated incident infections by self-report were excluded, as were studies that recruited participants directly from prisons or other detention settings. We did not exclude studies on the basis of study design or language, but included only studies reporting original results. We contacted authors of cohort studies that met the inclusion or exclusion criteria, but that did not report on the outcomes of interest, to request data. This approach followed methods used in previous systematic reviews on HIV and HCV.^18^, ^19^ Studies that presented data or provided unpublished data on the outcomes of interest were included. When there were multiple studies from the same cohort of PWID with estimates of the same outcome, only the most comprehensive study, in terms of the number of participants and years covered, was included. Methods of the analysis and inclusion criteria were specified in advance and documented in a protocol available on request.

### Data analysis

JS extracted data (list of data extracted in appendix) from included studies using Microsoft Excel 2016 for Mac; these were checked by HF, and discrepancies were resolved by PV. If not reported, effects and 95% CIs were calculated from raw data. We extracted or generated crude and adjusted incidence rate ratios and hazard ratios (HRs) from longitudinal studies that measured incident HIV or HCV infection. In studies that found no incident cases in the exposed or unexposed group, a fraction of a case (0·5 incident infections) was added to both groups before computing the incidence rate ratio. We extracted crude and adjusted odds ratios or relative risks (RRs) from cross-sectional studies that used biological markers (eg, the presence of HCV RNA in the absence of HCV antibody) of recent HIV or HCV infection. We transformed unadjusted and adjusted odds ratios and their 95% CIs to RRs when incidence was considered to be common (>10 per 100 person-years) using previously published methods.^20^

We assessed the risk of bias in each study for each outcome using the Newcastle–Ottawa Scale,^21^ in which a maximum of nine stars are awarded according to the selection of study groups, comparability of the groups, and ascertainment of the outcome of interest. The Newcastle–Ottawa Scale requires the selection of the most important potential confounders that should be controlled for in studies. We identified exposure to opioid substitution therapy, recent homelessness, and stimulant injecting as important potential confounders that should be adjusted for in our analyses. Opioid substitution therapy is associated with a reduced risk of HCV and HIV acquisition^18^, ^19^ and with a reduced prevalance of incarceration.^22^ Homelessness, and stimulant injecting (although studies generally consider only cocaine injecting) are positively associated with risk of HCV or HIV acquisition^23^, ^24^ and incarceration history.^13^, ^25^ In assessing the comparability of the groups, one star was awarded for adjusting for opioid substitution therapy exposure and one star for adjusting for recent homelessness, stimulant injecting, or both. Risk of bias for unpublished estimates was assessed by referring to the study methods in the corresponding published paper. For each outcome, publication bias of included studies was assessed with a funnel plot and Egger's test.^26^

To provide a summary estimate for each outcome, effect measures and their SEs were log-transformed, with unadjusted and adjusted estimates being pooled separately. Random effects meta-analysis was used to obtain summary effect measures because high between-study variability was expected. We evaluated between-study heterogeneity using the *I*^2^ statistic and the p value for heterogeneity (Cochran's *Q* statistic).^27^

Subgroup analyses and random-effects meta-regression analyses were done to explore potential sources of heterogeneity for outcomes that showed moderate to high heterogeneity between studies. All variables significant in univariable meta-regression (p≤0·05) were included in multivariable meta-regression; opioid substitution therapy coverage and proportion of participants homeless at baseline were included a priori in multivariable models. Sensitivity analyses were done to assess the effect of including in separate meta-analyses only: studies at low to moderate risk of bias (Newcastle–Ottawa Scale≥6), longitudinal studies, studies reporting HRs, and studies in which more than 90% of study participants were recent injectors (injected within past 6 months) at baseline. We also assessed the effect of excluding studies in which no incident infections were found in one exposure group. We did all the analyses using STATA version 14.

### Role of the funding source

The sponsors of the study had no role in study design, data collection, data analysis, data interpretation, or writing of the report. The corresponding author had full access to all the data in the study and had final responsibility for the decision to submit for publication.

## Results

**Figure 1:**
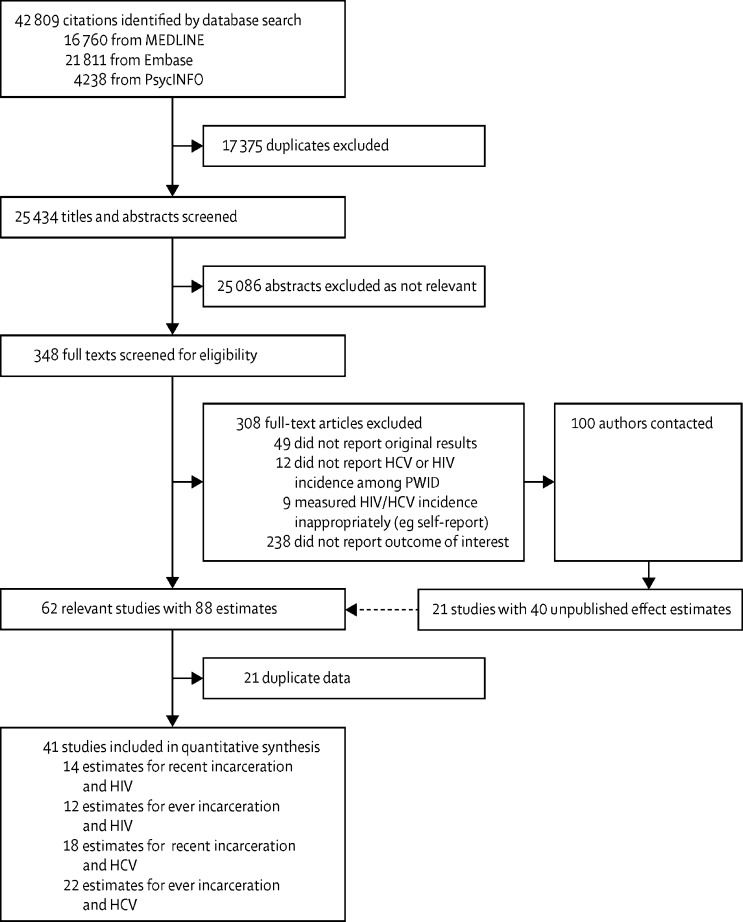
Study selection HCV=hepatitis C virus. PWID=people who inject drugs.

The searches identified 42 809 citations in total, of which 25 434 were unique records (figure 1). Initial screening of titles and abstracts excluded 25 086 records. Following the full-text screening of the remaining 348 records, seven by use of Google Translate, 40 records were deemed as meeting the inclusion criteria. In addition, we identified 90 studies of HIV or HCV incidence among PWID that met the inclusion criteria but did not report the outcomes of interest. The authors of these studies and investigators of other cohorts (ten) known to us who had not published their incidence data were contacted. Of these, unpublished data (40 different effect estimates) were obtained from 21 studies; 17 effect estimates were obtained from ongoing or completed studies that had not yet published their incidence data, 14 from studies with publications reporting HIV or HCV incidence but not reporting outcomes of interest, and nine were updates of previously published effect estimates that included more data. From the 62 (41 + 21) studies meeting the inclusion criteria, 21 (appendix) were excluded as multiple studies of the same cohort presenting data on the same outcome. One study published in non-English was included in the review; we obtained the dataset for this study directly and so did not depend on Google Translate to obtain the effect estimates or baseline characteristics.

The included studies (table) varied in average follow-up duration (longitudinal studies only 0·6–6·3 years), proportion of the sample who were women (0·0–59·8%), background HIV (0·0–34·2%) and HCV (18·6–82·0%) prevalence, HIV (0·1–25·3 per 100 person-years) and HCV (0·5–66 per 100 person-years) incidence, study period (1988–2016), and publication year (2000–17).

**Table:** Study details and outcomes available

	**Study period**	**Location (city, country)**	**Study design**	**Sample size**	**Definition of recent incarceration**	**Effect of recent incarceration**	**Effect of past incarceration**	**Confounders included in adjusted estimates**
Aladashvili et al (unpublished)	1997–2001	Tbilisi, Batumi, and Poti, Georgia	Cohort^28^, ^29^	1031	Past 12 months	HIV: IRR 2·6 (0·61–11·18); HCV: IRR 1·56 (0·57–4·23)	HIV: IRR 0·38 (0·02–6·23); HCV: IRR: 0·50 (0·12–2·01)	..
Azim (unpublished)	2003–07	Dhaka, Bangladesh	Cohort^30^	561	Past 6 months	HIV: IRR 0·64 (0·15–2·75); HCV: IRR 1·36 (0·60–3·09)	HIV: IRR 1·44 (0·42–4·94); HCV: IRR 2·04 (0·90–4·65)	..
Blome et al^31^	1997–2005	Malmo, Sweden	Cohort	332	NA	..	HCV: RR 1·27 (1·05–1·53); aRR 1·3 (1·06–1·49)	Intravenous heroin and amphetamine use; duration of intravenous use of amphetamines
Bruneau (unpublished)	1992–2008	Montreal, Canada	Cohort^32^	2137	Past 6 months	HIV: HR 1·88 (1·34–2·65); aHR 1·34 (0·94–1·89)	HIV: HR 1·57 (1·07–2·29); aHR 0·94 (0·63, 1·41)	Age; sex; unstable housing; cocaine use; heroin use; syringe sharing with people known to be HIV positive; repeatedly flushing and pulling back during injection; sex with people known to be HIV positive; recruitment period
Bruneau et al^33^	2004–13	Montreal, Canada	Cohort	226	Past 3 months	HCV reinfection: HR 0·94 (0·30–3·10); aHR 0·95 (0·30–3·30);	..	Method of HCV initial clearance; age; gender; cocaine injection; heroin injection; prescription opioid injection; sharing syringe or injection paraphernalia
Brunton et al^34^	1994–96	Auckland, New Plymouth, Wellington, Christchurch, and Dunedin, New Zealand	Cohort	44	Since last follow-up (on average 2 years apart)	HCV: IRR 2·75 (0·34–21·99)	..	..
Choopanya et al^35^	1995–98	Bangkok, Thailand	Cohort	1209	Since last visit (scheduled for every 3 months)	HIV: IRR 3·39 (2·35–4·90)	HIV: IRR 1·70 (1·09–2·65)	..
Craine et al^36^	2004–06	South Wales, UK	Cohort	286	Past 12 months	HCV: IRR 1·36 (0·48–3·85)	..	..
Dietze (unpublished)	2008–18	Melbourne, Australia	Cohort^37^	645	NA	..	HCV (primary and reinfection): IRR 0·73 (0·47–1·13)	..
Hagan et al^38^	2002–05	Baltimore, Seattle, New York, Los Angeles, and Chicago, USA	Cohort	483	NA	..	HCV: IRR 1·51 (0·81–2·8)	..
Havens (unpublished)	2008–11	Kentucky, USA	Cohort^39^	184	Past 6 months	HCV: IRR 2·80 (1·36–5·77)	HCV: IRR 1·12 (0·65–1·92)	..
Hellard (unpublished)	2005–10	Melbourne, Australia	Cohort^40^	413	NA	..	HCV: HR 0·52 (0·14–1·97)	..
Hope (unpublished)	2011–13	England, Wales, and Northern Ireland, UK	Cross-sectional	2816	NA	..	HCV: OR 1·16 (0·82–1·63); aOR 1·06 (0·75–1·51)	Cocaine injection; homelessness; prescribed treatment for drug use
Hope (unpublished)	2014–15	England, Wales, and Northern Ireland, UK	Cross-sectional	1932	NA	..	HCV: OR 1·74 (0·93–3·25); aOR 1·60 (0·85–3·03)	Cocaine injection; homelessness; prescribed treatment for drug use
Hu et al^41^	1996	Bangkok, Thailand	Cross-sectional	1488	NA	..	HIV: RR 1·65 (1·09–2·51)	..
Hutchinson (unpublished)	2008–13	Scotland, UK	Cross-sectional^42^	4783	Past 6 months	HCV: RR 2·02 (0·89–4·61); aRR 1·21 (0·51-2·84)	HCV: RR 1·44 (0·73–2·84); aRR 1·22 (0·61–2·44)	Homelessness; methadone use; injected crack or cocaine in past 6 months
Iversen (unpublished)	1995–2012	National, Australia	Cohort^43^	3490	Past 12 months	HIV: HR 2·16 (0·7–6·65)	HIV: HR 0·71 (0·24–2·12)	..
Iversen et al^44^	1995–2010	National, Australia	Cohort	724	Past 12 months	HCV: HR 2·84 (2·01–4·02); aHR 2·68 (1·88–3·83)	..	Last drug injected; daily injection; location; study period
Lucas et al^45^	2013	15 sites, India	Cross-sectional	14 481	Past 6 months	HIV: RR 2·07 (0·94–4·57)	..	..
Lucidarme et al^46^	1999–2001	Northern and eastern France, France	Cohort	165	NA	..	HCV: IRR 1·91 (0·71–5·13)	..
Maher (unpublished)	2012	Australia	Cross-sectional^47^	2391	Past 12 months	HCV: RR 2·31 (0·86–6·21); aRR 2·15 (0·74–5·22)	..	Exposure to OST or MMT; duration of injecting; cocaine use
Martin et al^48^	2005–12	Bangkok, Thailand	Cohort	2413	Past 3 months	HIV: HR 3·10 (1·70–5·60); aHR 2·70 (1·40–4·90)	..	Age; injection frequency; methamphetamine injection; shared needles; recently in police cell
Mehta (unpublished)	1988–2009	Baltimore, USA	Cohort^49^, ^50^	1983	Past 6 months	HIV: IRR 1·07 (0·8–1·42); aIRR 0.97 (0·72-1·31); HCV: IRR 1·60 (0·55–4·68); aIRR 2·39 (0·78–7·3)	..	Cocaine injection; heroin injection; methadone treatment; homelessness
Micallef et al^51^	1993–2002	Sydney, Australia	Cohort	423	NA	..	HCV: IRR 2·48 (1·72–3·59); HCV reinfection: IRR 1·56 (0·43–5·65)	..
Milloy (unpublished)	2005–16	Vancouver, Canada (ARYS)	Cohort^52^	476	Past 6 months	HCV: HR 2·61 (1·69–4·04); aHR 2·46 (1·56–3·86)	HCV: HR 1·24 (0·77–2·01); aHR 1·28 (0·77–2·13)	Methadone treatment; homelessness; cocaine injection
Milloy (unpublished)	1996–2016	Vancouver, Canada (VIDUS)	Cohort^53^	1763	Past 6 months	HIV: HR 1·80 (1·32–2·44); aHR 1·76 (1·28–2·41); HCV: HR 1·16 (0·81–1·68); aHR 1·12 (0·77–1·61)	HIV: HR 0·82 (0·58–1·15); aHR 0·86 (0·61–1·21); HCV: HR 0·75 (0·54–1·05); aHR 0·70 (0·50–0·98);	Methadone treatment; homelessness; cocaine injection
Morris et al^54^	2008–11	Sydney, Australia	Cohort	294	NA	..	HCV: IRR 0·33 (0·09–1·14)	..
Morris et al^54^	2004–11	Montreal, Canada	Cohort	244	NA	..	HCV: IRR 1·16 (0·68–1·96)	..
Morris et al^54^	2000–11	San Francisco, USA	Cohort	398	NA	..	HCV: IRR 1·11 (0·68–1·83)	..
Mravčík (unpublished)	2002–05	Nine regions, Czech Republic	Cohort^55^	173	NA	..	HCV: IRR 0·81 (0·26–2·5)	..
Platt, Hope and Hickman (unpublished)	2006–09	Bristol, Leeds, and Birmingham, UK	Cross-sectional 56, 57	1247	Past 12 months	HCV: RR 1·22 (0·48–3·09); aRR 1·21 (0·44–3·16)	..	OST exposure; homelessness; injecting duration; use of cocaine
Roy (unpublished)	2003–16	Eastern central Canada and Quebec and Ontario, Canada	Cohort^58^	1735	Past 6 months	HIV: HR 1·76 (1·05–2·96); aHR 1·77 (1·03–3·02); HCV: HR: 1·08 (0·78-1·49); aHR 0·93 (0·67-1·29)	..	Homelessness; OST exposure; using syringes used by someone else; cocaine most often injected drug; injecting daily; age; gender; prostitution; urban sites
Sacks-Davis et al^59^	2004–12	Montreal, Canada	Cohort	854	Past 3 months	HCV: HR 2·38 (1·43–3·95); aHR 1·98 (1·18–3·32)	..	Recent cocaine injection; recently injected at least daily; prescription opioid injection by area of residence
Smyrnov (unpublished)	2013	29 cities, Ukraine	Cross-sectional^60^	9502	Past 6 months	HIV: OR 1·91 (0·26–14·18)	HIV: OR 1·65 (0·76–3·59)	..
Smyth et al^61^	1992–99	Dublin, Ireland	Cohort	100	Since last visit	HCV: IRR 0·79 (0·42–1·48)	HCV: IRR 1·73 (1·03–2·89)	..
Spittal et al^62^	2003–09	Vancouver and Prince George, Canada	Cohort	148	Past 6 months	HCV: HR 1·25 (0·83–1·89)	HCV: HR 1·11 (0·59–2·09)	..
Strathdee (unpublished)	2006–10	Tijuana, Mexico (EC3)	Cohort^63^	1010	Past 6 months	HIV: IRR 0·84 (0·05–13·96)	HIV: HR 0·67 (0·25–1·85)	..
Strathdee (unpublished)	2011–16	Tijuana, Mexico (EC4)	Cohort^64^	737	Past 6 months	HIV: HR 0·67 (0·28–1·60)	HIV: HR 0·56 (0·25–1·26)	..
Suntharasamai et al^65^	1999–2003	Bangkok, Thailand	Cohort	2546	Past 6 months	HIV: HR 2·00 (1·40–2·70); aHR 1·40 (1·00–1·90)	..	Injection frequency; sharing needles; MMT use
Sypsa et al^66^	2012–13	Athens, Greece	Cohort	3320	NA	..	HIV: HR 2·4 (1·27–4·53); aHR 1·99 (0·98–3·85)	Age; gender; country of origin, homelessness; size of participants' injecting network; OST treatment; main substance of use; injecting drug use in past month; injecting frequency; sharing syringes; use of drugs shared between people with a used syringe
Tsui et al^67^	2000–13	San Francisco, USA	Cohort	552	Past 3 months	HCV: 1·58 (1·12–2·23)	..	..
Vallejo et al^68^	2001–06	Barcelona, Madrid, and Seville, Spain	Cohort	513	NA	..	HCV: IRR 1·20 (0·57–2·50)	..
Yen (unpublished)	2007–10	Taipei, Taiwan	Cohort^69^	236	NA	..	HIV: IRR 0·74 (0·04–14·4)	..

IRR=incidence rate ratio. HCV=hepatitis C virus. NA=not applicable. RR=relative risk. aRR=adjusted relative risk. HR=hazard ratio. aHR=adjusted hazard ratio. MMT=methadone maintenance treatment. aIRR=adjusted incidence rate ratio. OR=odds ratio. aOR=adjusted odds ratio. OST=opioid substitution therapy. ARYS=At Risk Youth Study. VIDUS=Vancouver Infection Drug Users Study. EC3=El Cuete Phase III. EC4=El Cuete Phase IV. Unpublished means that the estimates are unpublished.

**Figure 2:**
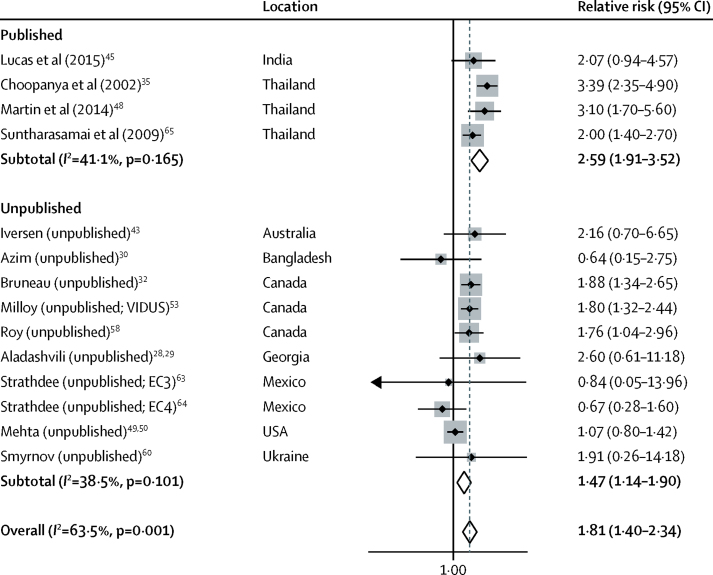
Meta-analysis of studies showing the crude effect of recent incarceration on the risk of HIV acquisition among people who inject drugs, by publication status. VIDUS=Vancouver Infection Drug Users Study. EC3=El Cuete Phase III. EC4=El Cuete Phase IV.

14 studies, including ten unpublished estimates, reported the effect of recent incarceration on HIV acquisition risk: 12 longitudinal studies and two cross-sectional. Definitions of recent incarceration in these studies included in the past 3 months, 6 months, or 12 months, or since last follow-up visit (scheduled for every 3 months; appendix). Recent incarceration was associated with an 81% increase in HIV acquisition risk (RR 1·81; 95% CI 1·40–2·34; p<0·0001; figure 2) with moderate heterogeneity between studies (*I*^2^=63·5%; p=0·001). Pooled effect estimates were higher across published studies (2·59; 1·91–3·52; p<0·0001) than across unpublished studies (1·47; 1·14–1·90; p=0·003; figure 2). The effect was reduced in studies that adjusted for confounders (1·48; 1·16–1·90; p=0·002) and in studies at low to moderate risk of bias (1·65; 1·26–2·16; p=0·006) but did not differ in other sensitivity analyses (appendix).

**Figure 3:**
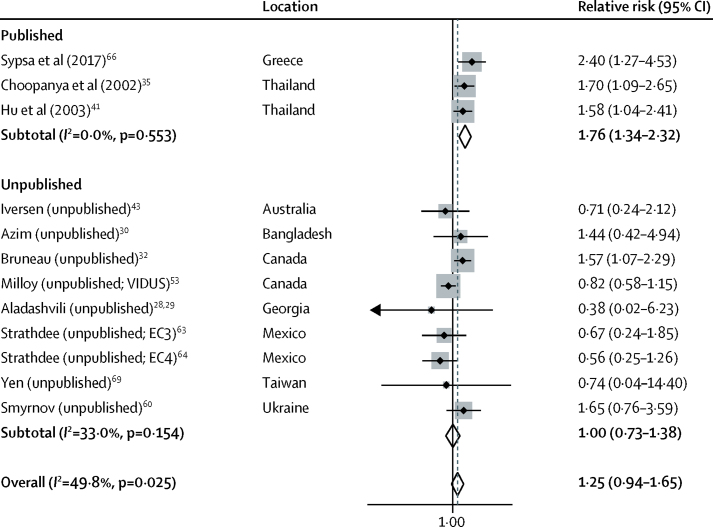
Meta-analysis of studies showing the crude effect of past incarceration on the risk of HIV acquisition among people who inject drugs, by publication status VIDUS=Vancouver Infection Drug Users Study. EC3=El Cuete Phase III. EC4=El Cuete Phase IV.

12 studies, including nine unpublished estimates, reported the effect of past incarceration on HIV acquisition risk: ten longitudinal studies and two cross-sectional. Past incarceration was not significantly associated with HIV acquisition risk (RR 1·25; 95% CI 0·94–1·65; p=0·112; figure 3) with moderate heterogeneity between studies (*I*^2^=49·8%; p=0·025). Pooled effect estimates were higher across published studies (RR 1·76; 95% CI 1·34–2·32; p<0·0001) than across unpublished studies (RR 1·00; 0·73–1·38; p=0·980). The effect was null among studies that adjusted for confounders (RR 1·06; 0·71–1·56; p=0·785), studies reporting only HRs (HR 1·05; 0·67–1·64; p=0·842), studies at low to moderate risk of bias (RR 1·06; 0·66–1·71; p=0·802), and studies in which at least 90% of participants were recent injectors at baseline (RR 0·96, 0·63–1·48, p=0·864; appendix).

In the meta-regression (appendix), there was no evidence that the effect of past or recent incarceration on HIV acquisition risk varied by geographical region or country income level. Published estimates of the effects of recent incarceration (ratio of RRs 1·76 [95% CI 1·12–2·76], p=0·018) and past incarceration (1·76 [1·05–2·92], p=0·034) were higher than unpublished estimates. The effect of recent incarceration was lower in studies involving older participants (mean or median age ≥34·3 years *vs* <34·3 years; ratio of RRs 0·54 [0·33–0·87], p=0·016), and the effect of past incarceration was lower in studies with a greater proportion of women (per 10% increase in the proportion of women; 0·78 [0·64–0·95], p=0·020) and in studies spanning 4 years or more (*vs* studies spanning <4 years; 0·56 [0·35–0·89], p=0·019). Multivariable meta-regression analyses did not show any of these associations, but are likely to be underpowered (appendix).

**Figure 4:**
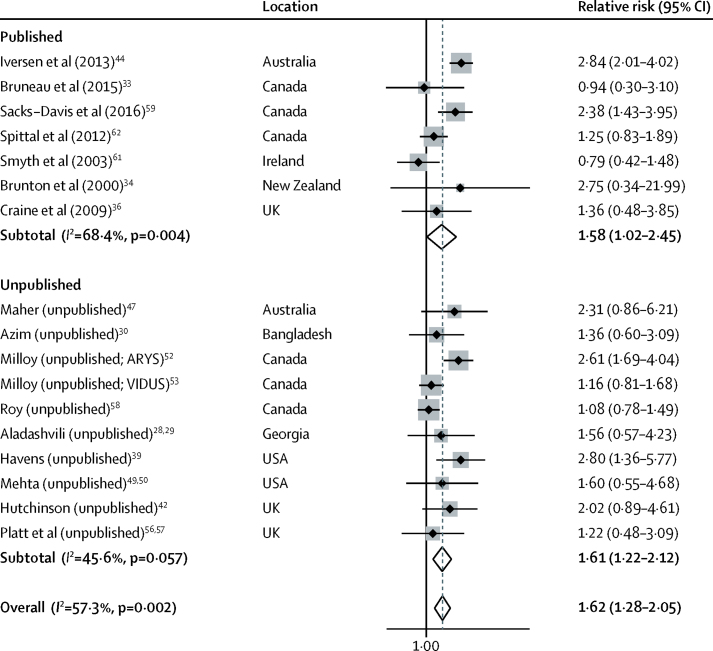
Meta-analysis of studies showing the crude effect of recent incarceration on the risk of hepatitis C virus acquisition among people who inject drugs, by publication status ARYS=At Risk Youth Study. VIDUS=Vancouver Infection Drug Users Study.

18 studies, including ten unpublished estimates, reported the effect of recent incarceration on HCV acquisition risk: 14 longitudinal studies and three cross-sectional. Definitions of recent incarceration included in the past 3 months, 6 months, or 12 months, or since last follow-up visit (on average every 2 years in one study; unknown in the other; appendix). Recent incarceration was associated with a 62% increase in HCV acquisition risk (RR 1·62; 95% CI 1·28–2·05; p<0·0001; figure 4), with moderate heterogeneity between studies (*I*^2^=57·3%; p=0·002). Pooled effect estimates were similar across published (RR 1·58; 95% CI 1·02–2·45; p=0·041) and unpublished (1·61; 1·22–2·12; p=0·001) estimates. The effect was the same in studies that adjusted for confounders (1·60; 1·21–2·11; p<0·0009) and did not differ in other sensitivity analyses (appendix).

**Figure 5:**
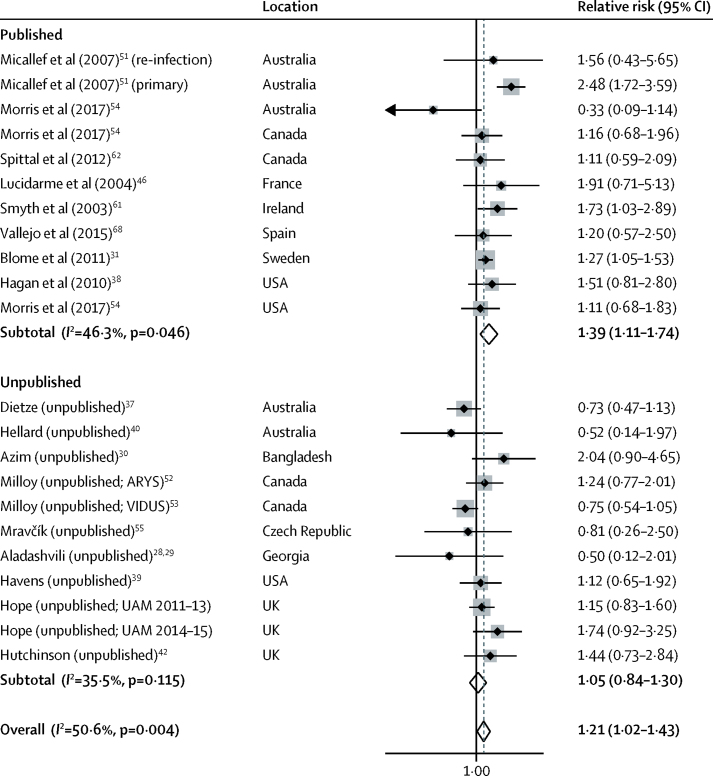
Meta-analysis of studies showing the crude effect of past incarceration on the risk of hepatitis C virus acquisition among people who inject drugs, by publication status ARYS=At Risk Youth Study. VIDUS=Vancouver Infection Drug Users Study. UAM=Unlinked Anonymous Monitoring survey of people who inject drugs.

22 studies, including 11 unpublished estimates, reported the effect of past incarceration on HCV acquisition risk. Past incarceration was associated with a 21% increase in HCV acquisition risk (RR 1·21; 95% CI 1·02–1·43; p=0·027; figure 5), with moderate heterogeneity between studies (*I*^2^=50·6%; p=0·004). Pooled effect estimates were higher in published studies (1·39; 1·11–1·74; p=0·004) than in unpublished studies (1·05; 0·84–1·30; p=0·680). In sensitivity analyses (appendix), the effect was not significant in studies that adjusted for confounders (1·12; 0·88–1·42; p=0·366), in those that had low to moderate risk of bias (0·96; 0·75–1·22; p=0·724), in those that reported only HRs (HR 0·92; 95% CI 0·68–1·25; p=0·595), and in those in which at least 90% of participants were recent injectors at baseline (RR 1·03; 95% CI 0·80–1·32; p=0·824).

In the univariable meta-regression, there was no evidence that the effect of past or recent incarceration on HCV acquisition risk varied by geographical region, country income level, or proportion of female participants (appendix). There was a greater effect for recent incarceration in countries with higher prevalences of incarceration (greater than or equal to the global average [144 per 100 000 population] or higher *vs* less than global average; ratio RRs 1·74 [95% CI 1·09–2·76], p=0·022; appendix). The effect of past incarceration was lower in more recent studies (midpoint of study October, 2005 or later *vs* earlier than October, 2005; ratio of RRs 0·69 [0·50–0·95], p=0·024; appendix). Multivariable meta-regression analyses did not show any of these associations, but are likely to be underpowered (appendix).

In univariable meta-regression (appendix) of the adjusted estimates of the effect of recent incarceration on HCV acquisition risk, there was no evidence that estimates adjusted for recent homelessness (ratio of RRs 0·66 [95% CI 0·37–1·17], p=0·132), or opioid substitution therapy exposure (0·68 [0·37–1·26], p=0·192), or recent stimulant injecting (0.90 [0·37–2·20], p=0·795) were lower than those not adjusting for these variables (appendix).

Visual inspection of the funnel plots (appendix) and Egger's tests (p≥0·507) found no evidence of publication bias. For the 66 estimates included in the meta-analyses, the most common rating on the Newcastle–Ottawa Scale was six (n=19) or seven (n=15) stars out of a possible nine, signifying a moderate risk of bias (higher ratings represent a lower risk of bias; appendix). The ratings varied from three (n=1) to nine (n=7) stars. The average rating did not vary across outcomes.

## Discussion

We found strong evidence that recent incarceration is associated with increased HIV and HCV acquisition risk among PWID. Recent incarceration was associated with an 81% and 62% increased risk of HIV and HCV acquisition, respectively. Past incarceration was only weakly associated with an increase in HCV acquisition risk and was not associated with an increase in HIV acquisition risk; the association with HCV acquisition was removed in adjusted analyses.

There was evidence that the effect of recent incarceration on HCV acquisition risk was greater in studies from countries with higher prevalences of incarceration and that the effect of recent incarceration on HIV acquisition risk was lower in studies with older participants. There was also evidence that the effects of recent incarceration on HCV acquisition risk were lower if adjusted for recent homelessness or exposure to opioid substitution therapy, although these results were not significant.

Our study strengthens previously published evidence through contacting a large number of authors of incidence studies with unpublished estimates, thereby minimising publication bias. Nonetheless, our study has several limitations.

Although the search was not limited by language or publication source, little data came from the countries with the largest populations of PWID.^5^ Furthermore, most included studies were from high-income countries (34 of 48), with no studies being included from low-income countries or the Middle East and Africa. Whereas we found moderate to high amounts of heterogeneity between studies, there was no evidence that this was explained by geographical region or country income level.

It is possible that the effect of past incarceration on HCV acquisition risk could partly be attributable to higher-risk PWID being more likely to be incarcerated. Similarly, some of the increased risk associated with recent incarceration could represent increased acquisition risk during or even before incarceration, rather than following release. The limited availability of data on HIV and HCV incidence among incarcerated PWID^17^ prevented any systematic comparisons between settings with low or high incidence in prisons. Importantly, however, when pooling estimates from Scotland and Australia, settings which both have documented low HCV incidence among incarcerated PWID,^70^, ^71^ we found evidence of a strong effect of recent incarceration on HCV acquisition risk (appendix), suggesting increased risk after release. Additionally, there is evidence from Australia for lower HCV incidence in continuously incarcerated PWID than among PWID that are released and re-incarcerated,^71^ also suggesting heightened risk associated with release.

Data on incarceration were scarce in the studies and so we were unable to consider whether the effect of incarceration history differed by type of incarceration, duration of most recent sentence, whether opioid substitution therapy was given in prison, or overall frequency or length of incarceration. Future studies should include further detail on these factors.

It is probable that our multivariable, meta-regression analyses, which were prespecified in our protocol, suffered from a lack of power owing to the number of studies included in the review. As such, the findings of these analyses should be interpreted with care—specifically, a lack of evidence for an association does not mean that there is a lack of an association. It is important that future studies are designed to investigate how incarceration elevates HIV and HCV acquisition risk and what influences the magnitude of this increased risk.

All of the studies included in this meta-analysis were observational in nature, with many having a high risk of bias and few adjusting their effect estimates for potential confounders. Importantly, there were still strong associations between recent incarceration and increased HCV and HIV acquisition risk when limiting the analysis to adjusted estimates, or to studies at low to medium risk of bias.

Other studies have synthesised available evidence for the effect of interventions on HIV or HCV acquisition risk among PWID,^19^, ^72^, ^73^ considered the effect of polydrug use on HIV acquisition risk,^23^ and assessed the effect of laws criminalising drug use on HIV prevention and treatment outcomes among PWID.^12^ To our knowledge, however, this study is the first to have quantitatively synthesised available evidence on the effect of incarceration on HIV or HCV acquisition risk among PWID. Our findings are consistent with studies that find incarceration is associated with relapse to injecting drug use^8^ and that recently incarcerated PWID exhibit increased injecting risk behaviour^10^, ^11^ and reduced access to harm reduction interventions^14^ compared with PWID who do not report recent incarceration. Our findings are also consistent with studies that indicate increased risk of drug-related mortality following release.^9^ Whereas a reduction in opioid tolerance during incarceration is a probable explanation for this, evidence that elevated risk of mortality can persist for a year after release^74^ also suggests a sustained period of unstable drug use following release.

Our findings suggest that incarceration is an important enhancer of HIV and HCV acquisition risk among PWID globally. The mechanisms through which this occurs are also likely to affect PWID who are already infected with HIV or HCV, further increasing the contribution of incarceration to elevating HIV and HCV transmission. Mathematical modelling considering these effects suggests that incarceration could be an important driver of HIV and HCV transmission among PWID in many settings.^11^, ^15^, ^16^, ^17^ Additionally, our analysis suggests that the effects of incarceration might be greater in countries with high prevalences of incarceration, providing further impetus for reducing the incarceration of PWID.

Both opioid substitution therapy and needle and syringe programmes have been shown to be effective at reducing HCV and HIV transmission among PWID in the community.^19^, ^72^, ^73^ Although evidence of the effectiveness of opioid substitution therapy and needle and syringe programmes at reducing HIV and HCV transmission among PWID in prisons is scare, there is evidence that prison-based opioid substitution therapy is associated with reduced injecting risk and increased treatment entry and retention following release,^75^ and that prison-based needle and syringe programmes are effective at reducing syringe sharing among PWID and do not encourage drug use or represent a threat to safety.^76^ It is therefore probable that prison-based harm reduction interventions, with effective linkage to services following release, could reduce the risk associated with incarceration, as suggested by recent modelling.^11^, ^17^ Despite this, most countries do not provide opioid substitution therapy and needle and syringe programmes within prisons.^77^

However, these interventions are unlikely to be sufficient to fully prevent the elevated risk associated with incarceration and the period after release. Incarceration is interlinked with many other social determinants of health, including poverty, unemployment, and homelessness, and so it is probable that addressing these factors will be important for reducing the elevated acquisition risk associated with recent incarceration. We found that the effects of recent incarceration on HCV acquisition risk were lower when estimates were adjusted for recent homelessness, although these results were not significant. Homelessness following release could be on the causal pathway or act in synergy with incarceration to further elevate HCV acquisition risk; thus, the provision of stable housing following release could reduce the effects of recent incarceration on acquisition risk. Further research is required to better elucidate the factors associated with incarceration that increase HIV and HCV acquisition risk, aiding the development of interventions to reduce these risks.

In conclusion, our study provides strong evidence that recent incarceration is associated with substantial increases in HIV and HCV acquisition risk among PWID. Owing to the high prevalences of incarceration among PWID, incarceration is likely to be an important driver of HCV and HIV transmission and transmission among PWID. Our findings add to the growing body of evidence for the harms associated with international drug policy, which result in many people who use drugs being incarcerated, and support calls for decriminalisation of illicit drug use and greater access to prison-based harm reduction, with linkage following release. However, it is likely that addressing many of the multiple social vulnerabilities experienced by PWID will also be required to fully reduce the risks associated with incarceration.
