# Supplementary material for: Incarceration history and risk of HIV and hepatitis C virus acquisition among people who inject drugs: a systematic review and meta-analysis
Source: Lancet Infect Dis. 2018 Dec;18(12):1397–409. doi: 10.1016/S1473-3099(18)30469-9 (PMC6280039; doi:10.1016/S1473-3099(18)30469-9)
Supplement: Supplementary appendix [file mmc1.pdf]

THE LANCET Infectious Diseases

Supplementary webappendix

This webappendix formed part of the original submission and has been peer reviewed. We post it as supplied by the authors.

Supplement to: Stone J, Fraser H, Lim AG, et al. Incarceration history and risk of HIV and hepatitis C virus acquisition among people who inject drugs: a systematic review and meta-analysis. *Lancet Infect Dis* 2018; published online Oct 29. http://dx.doi.org/10.1016/S1473-3099(18)30469-9.

Appendix

Prisma Checklist

Section/topic	#	Checklist item	Reported on page #
**TITLE**			
Title	1	Identify the report as a systematic review, meta-analysis, or both.	1
**ABSTRACT**			
Structured summary	2	Provide a structured summary including, as applicable: background; objectives; data sources; study eligibility criteria, participants, and interventions; study appraisal and synthesis methods; results; limitations; conclusions and implications of key findings; systematic review registration number.	2
**INTRODUCTION**			
Rationale	3	Describe the rationale for the review in the context of what is already known.	5
Objectives	4	Provide an explicit statement of questions being addressed with reference to participants, interventions, comparisons, outcomes, and study design (PICOS).	5
**METHODS**			
Protocol and registration	5	Indicate if a review protocol exists, if and where it can be accessed (e.g., Web address), and, if available, provide registration information including registration number.	7
Eligibility criteria	6	Specify study characteristics (e.g., PICOS, length of follow-up) and report characteristics (e.g., years considered, language, publication status) used as criteria for eligibility, giving rationale.	6
Information sources	7	Describe all information sources (e.g., databases with dates of coverage, contact with study authors to identify additional studies) in the search and date last searched.	5.6
Search	8	Present full electronic search strategy for at least one database, including any limits used, such that it could be repeated.	Appendix: 3-4
Study selection	9	State the process for selecting studies (i.e., screening, eligibility, included in systematic review, and, if applicable, included in the meta-analysis).	6
Data collection process	10	Describe method of data extraction from reports (e.g., piloted forms, independently, in duplicate) and any processes for obtaining and confirming data from investigators.	6,7
Data items	11	List and define all variables for which data were sought (e.g., PICOS, funding sources) and any assumptions and simplifications made.	Appendix: 4
Risk of bias in individual studies	12	Describe methods used for assessing risk of bias of individual studies (including specification of whether this was done at the study or outcome level), and how this information is to be used in any data synthesis.	7
Summary measures	13	State the principal summary measures (e.g., risk ratio, difference in means).	6.7
Synthesis of results	14	Describe the methods of handling data and combining results of studies, if done, including measures of consistency (e.g., I^2) for each meta-analysis.	7
Risk of bias across studies	15	Specify any assessment of risk of bias that may affect the cumulative evidence (e.g., publication bias, selective reporting within studies).	7
Additional analyses	16	Describe methods of additional analyses (e.g., sensitivity or subgroup analyses, meta-regression), if done, indicating which were pre-specified.	7
**RESULTS**			
Study selection	17	Give numbers of studies screened, assessed for eligibility, and included in the review, with reasons for exclusions at each stage, ideally with a flow diagram.	8,9
Study characteristics	18	For each study, present characteristics for which data were extracted (e.g., study size, PICOS, follow-up period) and provide the citations.	10-12, Appendix 12-21

Risk of bias within studies	19	Present data on risk of bias of each study and, if available, any outcome level assessment (see item 12).	Appendix: 6-11
Results of individual studies	20	For all outcomes considered (benefits or harms), present, for each study: (a) simple summary data for each intervention group (b) effect estimates and confidence intervals, ideally with a forest plot.	14, 15, 17, 18
Synthesis of results	21	Present results of each meta-analysis done, including confidence intervals and measures of consistency.	13-18
Risk of bias across studies	22	Present results of any assessment of risk of bias across studies (see Item 15).	19
Additional analysis	23	Give results of additional analyses, if done (e.g., sensitivity or subgroup analyses, meta-regression [see Item 16]).	13, 15, 16, 18,19
**DISCUSSION**		s	
Summary of evidence	24	Summarize the main findings including the strength of evidence for each main outcome; consider their relevance to key groups (e.g., healthcare providers, users, and policy makers).	19
Limitations	25	Discuss limitations at study and outcome level (e.g., risk of bias), and at review-level (e.g., incomplete retrieval of identified research, reporting bias).	19,20
Conclusions	26	Provide a general interpretation of the results in the context of other evidence, and implications for future research.	22
**FUNDING**			
Funding	27	Describe sources of funding for the systematic review and other support (e.g., supply of data); role of funders for the systematic review.	8

Search Strategy
Below is the search strategy utilised in MEDLINE to identify potentially relevant studies. Keywords are listed in regular type and Medical Subject Heading (MESH) terms in **bold.**

1. hepatitis C OR HCV OR **exp hepatitis C/**
2. HIV OR "human immunodeficiency virus" OR **exp HIV Seropositivity/** OR **exp HIV Seroprevalence/** OR **exp HIV Infections/** OR **exp HIV/**
3. 1 OR 2
4. IDU OR IDUs OR IVDU OR IVDUs OR PWID OR PWIDs
5. substance* OR drug*
6. inject* OR intravenous
7. abuse* OR depend* OR use* OR misus* OR addict*
8. 5 adj3 6
9. 5 adj3 7
10. **exp substance abuse, intravenous/**
11. 4 OR 8 OR 9 OR 10
12. prevalence OR incidence OR epidemiol* OR survey OR rapid assessment OR situation assessment OR situational assessment OR RAR OR Cohort OR surveillance OR seroprevalence OR seroincidence OR seroepidemiol* OR seroconv* OR screening OR **exp epidemiologic methods/** OR **exp epidemiologic studies/** OR **exp sentinel surveillance/** OR **exp seroepidemiologic studies/** OR **exp cohort studies/** OR **exp cross-sectional studies/** OR **exp longitudinal studies/** OR **exp follow-up studies/** OR **exp prospective studies/**
13. 3 AND 11 AND 12
14. Limit 13: Animals/ NOT (Humans/ AND Animals/)
15. 13 NOT 14

Below is the search strategy utilised in Embase to identify potentially relevant studies. Keywords are listed in regular type and Emtree terms in **bold.**

1. hepatitis C OR HCV OR **exp hepatitis C/** OR **exp Hepatitis C virus/**
2. HIV OR "human immunodeficiency virus" OR **exp Human immunodeficiency virus** OR **exp Human immunodeficiency virus infection/** OR **exp Human immunodeficiency virus prevalence/**
3. 1 OR 2
4. IDU OR IDUs OR IVDU OR IVDUs OR PWID OR PWIDs
5. substance* OR drug*
6. inject* OR intravenous
7. abuse* OR depend* OR use* OR misus* OR addict*
8. 5 adj3 6
9. 5 adj3 7
10. **exp intravenous drug abuse/**
11. 4 OR 8 OR 9 OR 10
12. prevalence OR incidence OR epidemiol* OR survey OR rapid assessment OR situation assessment OR situational assessment OR RAR OR Cohort OR surveillance OR seroprevalence OR seroincidence OR screening OR **exp seroepidemiology/** OR **exp seroprevalence/** OR **exp epidemiology/** OR **exp prevalence/** OR **exp epidemiological data/** OR **exp incidence/** OR **exp observational study/** OR **exp cohort analysis/**
13. 3 AND 11 AND 12
14. Limit 13: Animals/ NOT (Humans/ AND Animals/)
15. 13 NOT 14

Below is the search strategy utilised in PsycINFO to identify potentially relevant studies. Keywords are listed in regular type and Thesaurus terms in **bold.**

1. hepatitis C OR HCV OR **exp Hepatitis/**

2. HIV OR “human immunodeficiency virus” OR **exp HIV/**
3. 1 OR 2
4. IDU OR IDUs OR IVDU OR IVDUs OR PWID OR PWIDs
5. substance* OR drug*
6. inject* OR intravenous
7. abuse* OR depend* OR use* OR misus* OR addict*
8. 5 adj3 6
9. 5 adj3 7
10. **exp Intravenous Drug Usage/**
11. 4 OR 8 OR 9 OR 10
12. prevalence OR incidence OR epidemiol* OR survey OR rapid assessment OR situation assessment OR situational assessment OR RAR OR Cohort OR surveillance OR seroprevalence OR seroincidence OR screening OR **exp epidemiology /** OR **exp surveys/** OR **exp cohort analysis/** OR **exp longitudinal studies/** OR **exp followup studies/** OR **exp prospective studies/**
13. 3 AND 11 AND 12
14. Limit 13: Animals/ NOT (Humans/ AND Animals/)
15. 13 NOT 14

Data extraction

Data were extracted from included studies by JS using Microsoft Excel, and were checked by another author (HF), with any discrepancies being resolved by a third author (PV). Key characteristics extracted for each study included: study location, design and period; publication year; inclusion and exclusion criteria; recruitment methodology; methods used to assess incident HIV or HCV infection; mean/median duration of follow-up and rate of attrition; background HIV and HCV prevalence; proportion of study participants at baseline who were recent injectors, female, currently or recently homeless, and had recent or past incarceration; mean age and duration of injecting; population opiate substitution therapy (OST) coverage; number of incident infections and total follow-up time, both stratified by incarceration history (either recent or past); unadjusted and adjusted measures of effect with 95% confidence intervals.

Appendix Table 1: List of studies excluded due to duplicate data.

Author (Year)	Location	Study Period	Cohort Name	Sample Size	Outcomes in Paper	
Ahamad (2015) [1]	Vancouver, Canada	1996-2013	VIDUS	1,639	Recent (last 6 months) incarceration on HIV acquisition risk	1
Aitken (2008) [2]	Melbourne, Australia	2005-2007	Networks 2	105	Past incarceration on HCV acquisition risk	1
Allen (2012) [3]	Scotland	2008-2009	NESI	1,367	Past and recent incarceration (past 6 months) on HCV acquisition risk	2
Boileau (2005) [4]	Montreal, Canada	1992-2001	St Luc Cohort	1,602	Recent (past 6 months) incarceration on HIV acquisition risk	1
Bruneau (2012) [5]	Montreal, Canada	2004-2009	St Luc/Hep-co	246	Recent (past 6 months) incarceration on HCV acquisition risk	1
Craib (2003) [6]	Vancouver, Canada	1996-2001	VIDUS	941	Recent (past 6 months) incarceration on HIV acquisition risk	1
Cullen (2015) [7]	England, Wales, and Northern Ireland	2011	UAM	980	Past incarceration on HCV acquisition risk	1
Hahn (2002) [8]	San Francisco, USA	2000-2001	UFO	195	Recent incarceration (past 3 months) on HCV acquisition risk	1
Hope (2011) [9]	Bristol, England	2006		115	Recent (past year) incarceration on HCV acquisition risk	1
Hudgens (2002) [10]	Bangkok, Thailand	1995-1998	BMA	1,124	Recent (since past visit) incarceration on HIV acquisition risk	1
Kerr (2016) [11]	Vancouver, Canada	1996-2013	VIDUS	1,647	Recent (past 6 months) incarceration on HIV acquisition risk	1
Martin (2010) [12]	Bangkok, Thailand	1999-2003	AIDSVAX B/E HIV vaccine trial	2,546	Recent (past 6 months) incarceration on HIV acquisition risk	1
Montain (2016) [13]	Vancouver, Canada	1996-2013	VIDUS	1,639	Recent (past 6 months) incarceration on HIV acquisition risk	1
Morris (2017) [14]	Melbourne, Australia	2005-2011	Networks 2	119	Past incarceration on HCV acquisition risk	1
Nelson (2002) [15]	Baltimore, USA	1988-1998	ALIVE	1,846	Recent incarceration on HIV acquisition risk	1
Patrick (2001) [16]	Vancouver, Canada	1996-1999	VIDUS	155	Recent (past 6 months) incarceration on HCV acquisition risk	1
Spittal (2002) [17]	Vancouver, Canada	1996-2001	VIDUS	939	Recent (past 6 months) incarceration on HIV acquisition risk	1
Stone (2017) [18]	Scotland	2008-2013	NESI	1,717	Recent (past 6 months) incarceration on HCV acquisition risk	1
Strathdee (2001) [19]	Baltimore, USA	1988	ALIVE	1,874	Recent (past 6 months) incarceration on HIV acquisition risk	1
Tyndall (2003) [20]	Vancouver, Canada	1996-2000	VIDUS	940	Recent (past 6 months) incarceration on HIV acquisition risk	1
Vanichseni (2001) [21]	Bangkok, Thailand	1995-1998	BMA	1,124	Recent (since past visit) incarceration on HIV acquisition risk	1

Appendix Table 2: Risk of bias of studies for the effect of recent incarceration on HIV acquisition risk. Average score: 6.6

Author and Year	Representativeness of the exposed cohort	Selection of the non-exposed cohort	Ascertainment of exposure	Demonstration that outcome of interest was not present at the start of the study	Comparability of cohorts on the basis of the design or analysis	Assessment of outcome	Was follow-up long enough for outcomes to occur	Adequacy of follow up of cohorts	Total (/9)
Aladashvili (unpublished)	-	*	*	*	-	*	*	*	6
Azim (unpublished)	-	*	*	*	-	*	*	*	6
Bruneau (unpublished)	*	*	*	*	*	*	*	*	8
Choopanya 2002 [22]	-	*	*	*	-	*	*	*	6
Iversen (unpublished)	-	*	*	*	-	*	*	-	5
Lucas 2015 [23]	*	*	*	-	-	*	-	-	4
Martin 2014 [24]	*	*	*	*	*	*	*	-	7
Mehta (unpublished)	*	*	*	*	**	*	*	*	9
Milloy (unpublished - VIDUS)	*	*	*	*	**	*	*	*	9
Roy (unpublished)	-	*	*	*	**	*	*	*	8
Smyrnov (unpublished)	*	*	*	-	-	*	-	-	4
Strathdee (unpublished – EC3)	*	*	*	*	-	*	*	*	7
Strathdee (unpublished – EC4)	*	*	*	*	-	*	*	*	7
Suntharasamai 2009 [25]	-	*	*	*	*	*	*	*	7

Appendix Table 3: Risk of bias of studies for the effect of past incarceration on HIV acquisition risk. Average score: 6.4

Author and Year	Representativeness of the exposed cohort	Selection of the non-exposed cohort	Ascertainment of exposure	Demonstration that outcome of interest was not present at the start of the study	Comparability of cohorts on the basis of the design or analysis	Assessment of outcome	Was follow-up long enough for outcomes to occur	Adequacy of follow up of cohorts	Total (/9)
Aladashvili (unpublished)	*	*	*	*	-	*	*	*	7
Azim (unpublished)	-	*	*	*	-	*	*	*	6
Bruneau (unpublished)	*	*	*	*	*	*	*	*	8
Choopanya 2002[22]	*	*	*	*	-	*	*	*	7
Hu 2003[26]	-	*	*	-	-	*	-	-	3
Iversen (unpublished)	-	*	*	*	-	*	*	-	5

Milloy (unpublished - VIDUS)	*	*	*	*	**	*	*	*	9
Smyrnov (unpublished)	*	*	*	-	-	*	-	-	4
Strathdee (unpublished – EC3)	*	*	*	*	-	*	*	*	7
Strathdee (unpublished – EC4)	*	*	*	*	-	*	*	*	7
Sypsa 2017 [27]	*	*	*	*	**	*	*	*	9
Yen (unpublished)	-	*	*	*	-	*	*	-	5

Appendix Table 4: Risk of bias of studies for the effect of recent incarceration on HCV acquisition risk. Average score: 6.6

Author and Year	Representativeness of the exposed cohort	Selection of the non-exposed cohort	Ascertainment of exposure	Demonstration that outcome of interest was not present at the start of the study	Comparability of cohorts on the basis of the design or analysis	Assessment of outcome	Was follow-up long enough for outcomes to occur	Adequacy of follow up of cohorts	Total (/9)
Aladashvili (unpublished)	-	*	*	*	-	*	*	*	6
Azim (unpublished)	-	*	*	*	-	*	*	*	6
Bruneau 2015 [28]	-	*	*	*	*	*	*	-	6
Brunton 2000 [29]	-	*	*	*	-	*	*	*	6
Craine 2009 [30]	*	*	*	*	-	*	*	*	7
Havens (unpublished)	*	*	*	*	-	*	*	*	7
Hutchinson (unpublished)	-	*	*	-	**	*	-	-	5
Iversen 2013 [31]	-	*	*	*	*	*	*	-	6
Maher (unpublished)	-	*	*	-	*	*	-	-	4
Mehta (unpublished)	*	*	*	*	**	*	*	*	9
Milloy (unpublished - VIDUS)	*	*	*	*	**	*	*	*	9
Milloy (unpublished - ARYS)	-	*	*	*	**	*	*	*	8
Platt, Hope and Hickman (unpublished)	*	*	*	-	**	*	-	-	6
Roy (unpublished)	-	*	*	*	**	*	*	*	8
Sacks-Davis 2016 [32]	*	*	*	*	*	*	*	*	8
Smyth 2003 [33]	-	*	*	*	-	*	*	-	5
Spittal 2012 [34]	-	*	*	*	-	*	*	-	5
Tsui 2014 [35]	-	*	*	*	**	*	*	*	8

Appendix Table 5: Risk of bias of studies for the effect of past incarceration on HCV acquisition risk. Average score: 6.2

Author and Year	Representa-tiveness of the exposed cohort	Selection of the non-exposed cohort	Ascertainment of exposure	Demonstration that outcome of interest was not present at the start of the study	Comparability of cohorts on the basis of the design or analysis	Assessment of outcome	Was follow-up long enough for outcomes to occur	Adequacy of follow up of cohorts	Total (/9)
Aladashvili (unpublished)	*	*	*	*	-	*	*	*	7
Azim (unpublished)	-	*	*	*	-	*	*	*	6
Blome 2011 [36]	-	*	*	*	*	*	-	-	5
Dietze (unpublished) [37]	-	*	*	*	-	*	*	*	6
Hagan 2010 [38]	-	*	*	*	-	*	*	*	6
Havens (unpublished)	*	*	*	*	-	*	*	*	7
Hellard (unpublished)	-	*	*	*	-	*	*	*	6
Hope (unpublished, 2011-13)	*	*	*	-	**	*	-	-	6
Hope (unpublished, 2014-15)	*	*	*	-	**	*	-	-	6
Hutchinson (unpublished)	-	*	*	-	**	*	-	-	5
Lucidarme 2004 [39]	-	*	*	*	-	*	*	*	6
Micallef 2007 [40]	-	*	*	*	-	*	*	-	5
Micallef 2007 [40]	*	*	*	*	-	*	*	-	6
Milloy (unpublished - VIDUS)	*	*	*	*	**	*	*	*	9
Milloy (unpublished - ARYS)	-	*	*	*	**	*	*	*	8
Morris 2017 (UFO) [14]	*	*	*	*	-	*	*	*	7
Morris 2017 (St Luc)[14]	*	*	*	*	-	*	*	*	7
Morris 2017 (HITS-C/CU)[14]	*	*	*	*	-	*	*	*	7
Mravčík (unpublished)	*	*	*	*	-	*	*	-	6
Smyth 2003 [33]	-	*	*	*	-	*	*	-	5
Spittal 2012 [34]	-	*	*	*	-	*	*	-	5
Vallejo 2015 [41]	-	*	*	*	-	*	*	-	5

Appendix Table 6: Characteristics of included studies for the effect of recent incarceration on HIV acquisition risk.

Author and Year	Cohort Name, recruitment site and methods, inclusion criteria	Definition of recent incarceration	Effect Estimate
Aladashvili (unpublished)	1997-98: primarily out-of-treatment convenience sample of community PWID using advertising, participant referral and snowball sampling techniques. 2000-2001: referral of clinic patients. 1997-98: Injecting illicit drugs within the last six months, age: 18-65. 2000-2001: Age>18, history of IDU in the previous year.	Last 12 months	IRR: 2.6 (0.61-11.18)
Azim (unpublished)	NSP clients. Male, Age>= 15, injected drugs at least once in the last 2 months, member of the NSP of CARE Bangladesh, not changed living area in the last 6 months.	Last 6 months	IRR: 0.64 (0.15-2.75)
Bruneau (unpublished)	St Luc Cohort: Street-level recruitment or community programs. Age>=18, injected drugs within past 6 months.	Last 6 months	HR: 1.88 (1.34-2.65)<br>AHR: 1.34 (0.94-1.89)<br>Adjusted for: Age, gender, unstable housing, cocaine use in past month, heroin use in past month, sharing a syringe with a person known to be HIV +ve, booting, sex with someone known to be HIV +ve, period of recruitment.
Choopanya 2002 [22]	BMA: 15 Bangkok Metropolitan Administration drug treatment clinics. History of injection drug use, age: 18-50, attending any of 15 BMA drug treatment clinics, not known to be HIV-seropositive.	Since last visit	IRR: 3.39 (2.35-4.9)
Iversen (unpublished)	ANSPS: NSP sites, consenting NSP attendees.	Last 12 months	HR: 2.16 (0.7-6.65)
Lucas 2015 [23]	RDS. Age>=18, injecting drug use in the prior 2 years, verbal informed consent, presented valid recruitment coupon (except for seeds)	Last 6 months	RR: 2.07 (0.94-4.57)
Martin 2014 [24]	Bangkok Tenofovir Study: 17 drug-treatment clinics. HIV-negative individuals, age:20–60, injecting drugs during previous year. Excluded for hepatitis B virus surface antigen, pregnant or breastfeeding women.	Last 3 months	HR: 3.1 (1.7-5.6); AHR: 2.7 (1.4-4.9);<br>Adjusted for: age; injection frequency past 3 months; injected methamphetamine last 3 months; shared needles past 3 months; in police cell last 3 months.
Mehta (unpublished)	ALIVE: Community outreach. 1998-1999: non-medical injection-drug use within the preceding 11 years, age>18, free of AIDS. From 2005: no longer required to be AIDS-free at entry. 1994–1995: injected in past 3 years, 1998 and 2005–2008, injected in last year.	Last 6 months	IRR: 1.07 (0.8-1.42); AIRR: 0.97 (0.72-1.31);<br>Adjusted for: intravenous cocaine use; intravenous heroin use; methadone treatment; homelessness
Milloy (unpublished)	VIDUS: self-referral and street outreach from Vancouver's Downtown Eastside. Injected illicit drugs in the previous month, age>=18, resided in greater Vancouver.	Last 6 months	HR: 1.8 (1.32-2.44); AHR: 1.76 (1.28-2.41);<br>Adjusted for: current enrolment in methadone program, homeless in last 6 months, any cocaine injection in last 6 months
Roy (unpublished)	SurvUDI: NSP, drop-in centres, detention centres, detoxification clinics, and rehabilitation programmes, age>=14, injecting at least once within the past 6 months, speaking French or English, able to provide informed consent	Last 6 months	HR: 1.76 (1.05-2.96); AHR: 1.77 (1.03-3.02);<br>Adjusted for: homelessness, OST exposure, using syringes used by someone else, cocaine most often injected drug, injecting drugs every day, age, gender, prostitution, urban sites
Smyrnov (unpublished)	Aids Alliance IBBA: RDS.	Last 6 months	OR: 1.91 (0.26-14.18)
Strathdee (unpublished)	El Cuete III: RDS. Age>=18, injected illicit drugs within the past month (confirmed by inspection of injection stigmata), Spanish or English speaking; able to provide informed consent; no plans to permanently move out of the city in the next 18 months.	Last 6 months	IRR: 0.84 (0.05-13.96)
Strathdee (unpublished)	El Cuete IV: street-based outreach. Injecting illicit drugs in the past month (confirmed by visual assessment of 'track marks'), residing in Tijuana; speaking Spanish or English, age>=18.	Last 6 months	HR: 0.67 (0.28-1.6)
Suntharasamai 2009 [25]	AIDSVAX B/E HIV vaccine trial: 17 drug-treatment clinics, operated by the Bangkok Metropolitan Administration. Completion of informed consent, possession of a Thai national identification card, age: 20–60, availability for 3 years follow-up, injection drug use in the previous year, willingness to receive HIV counselling and testing, not known to be HIV positive.	Last 6 months	HR: 2.0 (1.4-2.7); AHR: 1.4 (1-1.9);<br>Adjusted for: Injection frequency during the 6 months before visit; sharing needles during the 6 months before visit; participated in methadone maintenance during the 6 months before visit.

Appendix Table 7: Characteristics of included studies for the effect of past incarceration on HIV acquisition risk.

Author and Year	Cohort Name, recruitment site and methods, inclusion criteria	Exposure to past incarceration	Effect Estimate
Aladashvili (unpublished)	1997-98: primarily out-of-treatment convenience sample of community PWID using advertising, participant referral and snowball sampling techniques. 2000-2001: referral of clinic patients. 1997-98: injecting illicit drugs within the last six months, 18<=age<=65. 2000-2001: age>18, history of IDU in the previous year.	Baseline	IRR: 0.38 (0.02-6.23)
Azim (unpublished)	NSP clients. Male, age>= 15, injected drugs at least once in the last 2 months, member of the NSEP of CARE Bangladesh, not changed living area in the last 6 months.	Measured at first follow-up	IRR: 1.44 (0.42-4.94)
Bruneau (unpublished)	St Luc Cohort: Street-level recruitment or community programs. Age>=18, injected drugs within past 6 months.	Baseline	HR: 1.57 (1.07-2.29)<br>AHR: 0.94 (0.63, 1.41)<br>Adjusted for: Age, gender, unstable housing, cocaine use in past month, heroin use in past month, sharing syringe with someone known to be HIV +ve, booting, sex with someone known to be HIV +ve, period of recruitment.
Choopanya 2002[22]	BMA: 15 Bangkok Metropolitan Administration drug treatment clinics. History of injection drug use, age: 18-50, attending any of 15 BMA drug treatment clinics, not known to be HIV-seropositive.	Time varying measured at preceding visit	IRR: 1.7 (1.09-2.65)
Hu 2003[26]	BMA: 15 Bangkok Metropolitan Administration drug treatment clinics. History of injection drug use, age: 18-50, attending any of 15 BMA drug treatment clinics, not known to be HIV-seropositive.	Baseline	RR: 1.65 (1.09-2.51)
Iversen (unpublished)	ANSPS: NSP sites, consenting NSP attendees.	Baseline	HR: 0.71 (0.24-2.12)
Milloy (unpublished)	VIDUS: self-referral and street outreach from Vancouver's Downtown Eastside. Injected illicit drugs in the previous month, age>=18, resided in greater Vancouver.	Baseline	HR: 0.82 (0.58-1.15);<br>AHR: 0.86 (0.61-1.21)<br>Adjusted for: current enrolment in methadone program; homeless in last 6 months; any cocaine injection in last 6 months
Smyrnov (unpublished)	Aids Alliance IBBA: RDS.	Baseline	OR: 1.65 (0.76-3.59)
Strathdee (unpublished)	El Cuete III: RDS. Age>=18, injected illicit drugs within the past month (confirmed by inspection of injection stigmata), Spanish or English speaking; able to provide informed consent; no plans to permanently move out of the city in the next 18 months.	Baseline	HR: 0.67 (0.25-1.85)
Strathdee (unpublished)	El Cuete IV: street-based outreach. Injecting illicit drugs in the past month (confirmed by visual assessment of 'track marks'), residing in Tijuana; speaking Spanish or English, age>=18.	Baseline	HR: 0.56 (0.25-1.26)
Sypsa 2017 [27]	ARISTOTLE: RDS. Seeds were recruited by staff of the Greek Organization Against Drugs. Valid RDS coupon, injected drugs without prescription in the past 12 months, age ≥18, resided in the Athens metropolitan area and had not participated previously in the current sampling round.	Time varying	HR: 2.4 (1.27-4.53)<br>AHR: 1.99 (0.98-3.85).<br>Adjusted for: age, sex; country of origin, currently homeless; size of participants network; currently in an OST program; main substance of use; injecting drug use in past month; frequency of injecting drug use; sharing syringes; use of drugs divided with a used syringe.

Author and Year	Cohort Name, recruitment site and methods, inclusion criteria	Exposure to past incarceration	Effect Estimate
Yen (unpublished)	MMT program at Taipei City Hospital. History of injecting drug use prior to enrolling in methadone maintenance.	Baseline	IRR: 0.74 (0.04-14.4)

Appendix Table 8: Characteristics of included studies for the effect of recent incarceration on HCV acquisition risk. All studies, except those marked with an asterisk, measured the effect on primary HCV infection. Those marked with an asterisk measured the effect on HCV reinfection.

Author and Year	Cohort Name, recruitment site and methods, inclusion criteria	Definition of recent incarceration	Effect Estimate
Aladashvili (unpublished)	1997-98: primarily out-of-treatment convenience sample of community PWID using advertising, participant referral and snowball sampling techniques. 2000-2001: referral of clinic patients, 1997-98: injecting illicit drugs within the last six months, age:18-65. 2000-2001: age>18, history of IDU in the previous year.	Last 12 months	IRR: 1.56 (0.57-4.23)
Azim (unpublished)	NSP clients. Male, age>= 15, injected drugs at least once in the last 2 months, member of the NSEP of CARE Bangladesh, not changed living area in the last 6 months.	Last 6 months	IRR: 1.36 (0.6-3.09)
Bruneau 2015 * [28]	HEPCO: Community based outreach. Injected drugs in last 6 months, age>=18, reside on the Island of Montréal.	Last 3 months	HR: 0.94 (0.3-3.1); AHR: 0.95 (0.3-3.3); Adjusted for: method of HCV initial clearance; age; gender; cocaine injection past month; heroin injection past month; prescription opioid injection past month; sharing syringes or injection paraphernalia past 6 months
Brunton 2000 [29]	Community, NSP and drug treatment. Snowball. Injected in last 12 months.	Since last visit	IRR: 2.75 (0.34-21.99)
Craine 2009 [30]	Treatment services, NSP, homeless hostels, street. Current or recent drug injector.	Last 12 months	IRR: 1.36 (0.48-3.85)
Havens (unpublished)	SNAP: RDS. Seeds had a lifetime history of injection drug use and recruited their drug-using peers. Age>=18, resided in Appalachian Kentucky, used prescription opioids, cocaine, heroin, or methamphetamine to get high in prior 30 days. Data analysis excludes never injectors at baseline.	Last 6 months	IRR: 2.8 (1.36-5.77)
Hutchinson (unpublished)	NESI: sites providing sterile injecting equipment across mainland Scotland. Ever injected drugs.	Last 6 months	RR: 2.02 (0.89-4.61); ARR: 1.21 (0.51-2.84) Adjusted for: homeless in last 6 months; currently on methadone; injected crack or cocaine in last 6 months
Iversen 2013 [31]	ANSPS: NSP sites, Consenting NSP attendees. Participated in the ANSPS in consecutive years and those with only a 1-year gap between participation records.	Last 12 months	HR: 2.84 (2.01-4.02); AHR: 2.68 (1.88-3.83) Adjusted for: last drug injected; daily injection; location; study period.
Maher (unpublished)	ANSPS: NSP Sites, consenting NSP attendees.	Last 12 months	RR: 2.31 (0.86-6.21); ARR: 2.15 (0.74-5.22) Adjusted for: exposure to OST/MMT; duration of injecting; use of crack
Mehta (unpublished)	ALIVE: Community outreach. 1998-1999: non-medical injection-drug use within the preceding 11 years, age>18, free of AIDS. From 2005 no longer required to be AIDS-free at entry. 1994–1995, injected in past 3 years, 1998 and 2005–2008, injected in last year.	Last 6 months	IRR: 1.6 (0.55-4.68); AIRR: 2.39 (0.78-7.3) Adjusted for: intravenous cocaine use; intravenous heroin use; methadone treatment; homeless
Milloy (unpublished)	ARYS: Snowball sampling and extensive street-based outreach. Age:14-26, use of drugs other than marijuana in the past 30 days. For analyses: first record where a participant reported injection in the last 6 months was set as baseline.	Last 6 months	HR: 2.61 (1.69-4.04); AHR: 2.46 (1.56-3.86)

Author and Year	Cohort Name, recruitment site and methods, inclusion criteria	Definition of recent incarceration	Effect Estimate
			Adjusted for: current enrolment in methadone program, homeless in last 6 months, any cocaine injection in last 6 months
Milloy (unpublished)	VIDUS: self-referral and street outreach from Vancouver's Downtown Eastside. Injected illicit drugs in the previous month, age>=18, resided in greater Vancouver.	Last 6 months	HR: 1.16 (0.81-1.68); AHR: 1.12 (0.77-1.61) Adjusted for: current enrolment in methadone program, homeless in last 6 months, any cocaine injection in last 6 months
Platt, Hope and Hickman (unpublished)	RDS, injected last 4 weeks	Last 12 months	RR: 1.22 (0.48-3.09); ARR: 1.21 (0.44-3.16) Adjusted for: exposure to OST, homeless in last 12 months, duration of injecting; use of Crack
Roy (unpublished)	SurvUDI: NSP, drop-in centres, detention centres, detoxification clinics, and rehabilitation programmes, age>=14, injecting at least once within the past 6 months, speaking French or English, able to provide informed consent	Last 6 months	HR: 1.08 (0.78-1.49); AHR: 0.93 (0.67-1.29) Adjusted for: homelessness, OST exposure, using syringes used by someone else, cocaine most often injected drug, injecting drugs every day, age, gender, prostitution, urban sites
Sacks-Davis 2016 [32]	HEPCO: Community based outreach. Injected drugs in last 6 months, age>=18, reside on the Island of Montreal. For data analysis, interviews excluded if invalid postal code or postal code associated with residence outside the Island of Montreal or a correctional facility.	Last 3 months	HR: 2.38 (1.43-3.95); AHR: 1.98 (1.18-3.32) Adjusted for: recent cocaine injection; recently injected at least daily; prescription opioid injection by area of residence.
Smyth 2003 [33]	Trinity Court drug treatment centre. History of injecting	Since last visit	IRR: 0.79 (0.42-1.48)
Spittal 2012 [34]	Cedar Project: Referral by health care providers, community outreach and word of mouth. Age: 14 -30 years of age, smoked illicit drugs in the last week or injected illicit drugs in the last month.	Last 6 months	HR: 1.25 (0.83-1.89)
Tsui 2014 [35]	UFO: Recruitment by outreach and word of mouth. Age<=30, injecting drugs in the prior month, English as primary language, and if recruited in 2003 or later, did not plan to travel outside of San Francisco within the next 3 months.	Last 3 months	AHR: 1.58 (1.12-2.23) Adjusted for: drug treatment past 3 months; age; duration injecting; gender; ethnicity; homeless past 3 months

Appendix Table 9: Characteristics of included studies for the effect of past incarceration on HCV acquisition risk. All studies, except those marked with an asterisk or dagger, measured the effect on primary HCV infection. Those marked with an asterisk measured the effect on HCV reinfection; those marked with a dagger measure the effect on primary HCV infection and HCV reinfection.

Author and Year	Cohort Name, recruitment site and methods, inclusion criteria	Exposure to past incarceration	Effect Estimate
Aladashvili (unpublished)	1997-98: primarily out-of-treatment convenience sample of community PWID using advertising, participant referral and snowball sampling techniques. 2000-2001: referral of clinic patients. 1997-98: Injecting illicit drugs within the last six months, age:18-65. 2000-2001: age>18, history of IDU in the previous year.	Baseline	IRR: 0.5 (0.12-2.01)
Azim (unpublished)	NSP. Male, age>= 15, injected drugs at least once in the last 2 months, member of the NSEP of CARE Bangladesh, not changed living area in the last 6 months.	Measured at first follow-up	IRR: 2.04 (0.9-4.65)
Blome 2011 [36]	New NSP participants, self-reported injection drug use, age>=20, signs of recent venepuncture and consent to HIV testing.	Baseline	RR: 1.27 (1.05-1.53); ARR: 1.3 (1.06-1.49); Adjusted for: intravenous heroin and amphetamine use; duration of intravenous use of amphetamines.
Dietze (unpublished)[†]	Melbourne Injecting Drug Users Cohort Study (MIX): recruited in Urban Melbourne through respondent-driven sampling (RDS), street outreach and snowball sampling. Age >=18 years; injected either heroin or methamphetamine at least six times over the previous six months; currently residing in Melbourne; willing to provide detailed contact information; able and willing to provide a valid Medicare card number. Two further eligibility criteria that aimed to recruit participants who were young (aged <31 years) and not prescribed OST were withdrawn during early recruitment.	Baseline	IRR: 0.73 (0.47-1.13)
Hagan 2010 [38]	DUIT: Street outreach, advertising, and coupon-based participant referrals. Injected illicit drugs in past 6 months, reside in recruitment city with no plans to move within 12 months, English-speaking, age:15–30, seronegative for HIV and HCV antibody. Data analysis excludes those not injecting during the follow-up period	Baseline	IRR: 1.51 (0.81-2.8)
Havens (unpublished)	SNAP: RDS. Seeds had a lifetime history of injection drug use and recruited their drug-using peers. Age>=18, resided in Appalachian Kentucky, used prescription opioids, cocaine, heroin, or methamphetamine to get high in the prior 30 days. Data analysis excludes never injectors at baseline.	Baseline	IRR: 1.12 (0.65-1.92)
Hellard (unpublished)	NETWORKS2: 3 major street drug markets across metropolitan Melbourne. Social networks approach. Injected in the previous six months	Baseline	HR: 0.52 (0.14-1.97)
Hope (unpublished)	UAM 2011-13: specialist drug services. Ever injected.	Baseline	OR: 1.16 (0.82-1.63); AOR: 1.06 (0.75-1.51); Adjusted for: injected crack last 4 weeks; homeless in the last year; currently prescribed treatment for drug use.
Hope (unpublished)	UAM 2014-15: specialist drug services. Ever injected.	Baseline	OR: 1.74 (0.93-3.25); AOR: 1.60 (0.85-3.03); Adjusted for: injected crack last 4 weeks; homeless in the last year; currently prescribed treatment for drug use.
Hutchinson (unpublished)	NESI: sites providing sterile injecting equipment across mainland Scotland. Ever injected drugs.	Baseline	RR: 1.44 (0.73-2.84); ARR: 1.22 (0.61-2.44); Adjusted for: homeless in last 6 months; currently on methadone; injected crack or cocaine in last 6 months
Lucidarme 2004 [39]	6 care centres in Northern and Eastern France, injected drugs at least once in their lifetime, HCV serology presumed to be negative.	Baseline	IRR: 1.91 (0.71-5.13)
Micallef 2007 [40]	Kirketon Road Centre primary health care facility. Anti-HCV antibody negative IDUs on initial testing from July 1993 who underwent repeat anti-HCV antibody testing prior to March 2002.	Baseline	IRR: 2.48 (1.72-3.59)

Author and Year	Cohort Name, recruitment site and methods, inclusion criteria	Exposure to past incarceration	Effect Estimate
Micallef 2007* [40]	Kirketon Road Centre. Subjects from the HCV seronegative cohort who seroconverted to anti-HCV antibody positive within 2 years of their most recent anti-HCV antibody negative result.	Baseline	IRR: 1.56 (0.43-5.65)
Milloy (unpublished)	ARYS: Snowball sampling and extensive street-based outreach. Age:14-26, use of drugs other than marijuana in the past 30 days. For analyses: first record where a participant reported injection in the last 6 months was set as baseline.	Baseline	HR: 1.24 (0.77-2.01); AHR: 1.28 (0.77-2.13); Adjusted for: current enrolment in methadone program, homeless in last 6 months, any cocaine injection in last 6 months
Milloy (unpublished)	VIDUS: self-referral and street outreach from Vancouver's Downtown Eastside. Injected illicit drugs in the previous month, age>=18, resided in greater Vancouver.	Baseline	HR: 0.75 (0.54-1.05); AHR: 0.7 (0.5-0.98); Adjusted for: current enrolment in methadone program, homeless in last 6 months, any cocaine injection in last 6 months"
Morris 2017 [14]	HITS-C & CU: Community-based outreach; open enrolment. Injection drug use within past 12 months, age>=16, anti-HCV negative.	Baseline	IRR: 0.33 (0.09-1.14)
Morris 2017 [14]	St Luc (HEPCO): Community-based outreach; open enrolment, Injection drug use in past 6 months, age≥14, anti-HCV negative.	Baseline	IRR: 1.16 (0.68-1.96)
Morris 2017 [14]	UFO: outreach word of mouth. Injection drug use in past month; age<30; anti-HCV negative.	Baseline	IRR: 1.11 (0.68-1.83)
Mravčík (unpublished)	Clients of the low-threshold facilities were used as seeds for snowball sampling. Injection drug use in the last 12 months, age>=15. Excluded for previous participation in study, and/or current participation in an abstinence-oriented or substitution treatment program, and/or an inability to understand the questions/instructions.	Baseline	IRR: 0.81 (0.26-2.5)
Smyth 2003 [33]	Trinity Court drug treatment centre. History of injecting	Baseline	IRR: 1.73 (1.03-2.89)
Spittal 2012 [34]	Cedar Project: Referral by health care providers, community outreach and word of mouth. Age: 14-30 years of age, smoked illicit drugs in the last week or injected illicit drugs in the last month, including crystal methamphetamine, crack-cocaine, heroin, or cocaine prior to enrolment.	Time varying	HR: 1.11 (0.59-2.09)
Vallejo 2015 [41]	Heroin-Itínere cohort: Street recruitment, targeted sampling, and chain-referral. Age<=30, heroin use at least 12 days in the past 12 months and at least 1 day in the past 3 months.	Measured during last period of follow up	IRR: 1.2 (0.57-2.5)

Appendix Table 10: Characteristics of all included studies. N/A = not available.

	Mean/median follow-up (years)	% female	Baseline prevalence		Incidence (/100 py)	
			HCV	HIV	HCV	HIV
Aladashvili (unpublished)	1.8	0.3	59.3	0.7	14.9	1.2
Azim (unpublished)	3.6	0	66.8	5.9	7.8	1.1
Blome 2011 [36]	1.5	21.4	60	0	38.3	N/A
Bruneau (unpublished)	2.1	19.5	N/A	10.5	N/A	3.3
Bruneau 2015 [28]	3	24	N/A	N/A	4.8	N/A
Brunton 2000 [29]	2	40.9	64	N/A	13	N/A
Choopanya 2002 [22]	2.4	6.5	N/A	29.9	N/A	5.8
Craine 2009 [30]	1	29.02	26.3	N/A	5.9	N/A
Dietze (unpublished)	3.4	32.6	45.8	0.2	9.0	N/A
Hagan 2010 [38]	0.67	32.9	34.4	N/A	17.3	N/A
Havens (unpublished)	4.7	44	53.3	N/A	12.6	N/A
Hellard (unpublished)	1	34.5	61.3	N/A	13.0	N/A
Hope (unpublished - UAM 2011-13)	Cross-sectional study	25.46	48.4	N/A	12.3	N/A
Hope (unpublished - UAM 2014-15)	Cross-sectional study	25.31	52.7	N/A	7.8	N/A
Hu 2003 [26]	Cross-sectional study	8.3	N/A	30.2	N/A	17.3
Hutchinson (unpublished)	Cross-sectional study	N/A	53.4	N/A	10.1	N/A
Iversen (unpublished)	N/A	N/A	N/A	0.95	N/A	0.1
Iversen 2013 [31]	1.5	37.4	53.6	N/A	17	N/A
Lucas 2015 [23]	Cross-sectional study	7.2	N/A	18.1	N/A	2.9
Lucidarme 2004 [39]	N/A	17.6	21.4	N/A	9	N/A
Maher (unpublished)	Cross-sectional study	31.5	53	N/A	42	N/A
Martin 2014 [24]	4	20.3	N/A	11	N/A	0.6
Mehta (unpublished)	6.6	24.1	N/A	21.4	17	2.1
Micallef 2007 (primary) [40]	1	59.8	N/A	N/A	17	N/A
Micallef 2007 (re-infection) [40]	1.2	55.6	N/A	N/A	42	N/A
Milloy (unpublished - VIDUS)	6.3	34.49	82	30.3	N/A	N/A
Milloy (unpublished -ARYS)	N/A	33	18.6	0.5	N/A	N/A
Morris 2017 (Sydney) [14]	N/A	32	N/A	N/A	21.4	N/A
Morris 2017 (San Francisco) [14]	N/A	19	N/A	N/A	23.5	N/A
Morris 2017 (Montreal) [14]	N/A	33	N/A	N/A	24.7	N/A
Mravčík (unpublished)	0.81	39.5	29.7	N/A	11.9	N/A
Platt, Hope and Hickman (unpublished)	Cross-sectional study	20.21	55.3	N/A	24.2	N/A
Roy (unpublished)	3.6	24.7	60.4	11.9	22.5	1.4
Sacks-Davis 2016 [32]	2.77	18	68.5	N/A	16.2	N/A
Smyrnov (unpublished)	Cross-sectional study	23.6	N/A	17.89	N/A	0.7
Smyth 2003 [33]	1	34	N/A	N/A	66	N/A

	Mean/median follow-up (years)	% female	Baseline prevalence		Incidence (/100 py)	
			HCV	HIV	HCV	HIV
Spittal 2012 [34]	2.6	53.4	46.4	N/A	11.6	N/A
Strathdee (unpublished - EC3)	2.1	13.16	N/A	4.46	N/A	0.8
Strathdee (unpublished - EC4)	3.7	38.1	N/A	3.54	N/A	0.9

Suntharasamai 2009 [25]	2.7	6.6	N/A	34.2	N/A	3.4
Sypsa 2017 [27]	0.7	15.45	N/A	15.1	N/A	4.5
Tsui 2014 [35]	N/A	31.9	39.2	4.4	25.1	N/A
Vallejo 2015 [41]	2.2	26.32	73.3	26.3	39.8	N/A
Yen (unpublished)	1.1	8.9	N/A	13.4	N/A	1.1

Appendix Table 11: Sensitivity analyses for the meta-analysis results. Pooled estimates of the crude and adjusted effect estimates shown for comparison.

	Number of studies	Effect size (95% CI)	P-value	I^2	P-value for heterogeneity
**Recent incarceration on HIV acquisition risk**					
Crude effect estimates	14	1.81 (1.40-2.34)	<0.001	63.5	0.001
Adjusted effect estimates	6	1.48 (1.16-1.90)	0.002	60.9	0.025
Published estimates only	4	2.59 (1.91-3.52)	<0.001	41.1	0.165
Unpublished estimates only	10	1.47 (1.14-1.90)	0.003	38.5	0.101
Only studies with low/medium risk of bias	8	1.65 (1.26-2.16)	0.006	64.7	0.006
Only longitudinal studies	12	1.79 (1.36-2.36)	<0.001	69.0	<0.001
Only hazard ratios	7	1.87 (1.52-2.31)	<0.001	29.0	0.207
Excluding studies with zero cases	13	1.82 (1.41-2.36)	<0.001	66.1	<0.001
Only studies with >90% injecting in last 6 months	7	1.72 (1.40-2.13)	<0.001	21.2	0.268
**Past incarceration on HIV acquisition risk**					
Crude effect estimates	12	1.25 (0.94-1.65)	0.112	49.8	0.025
Adjusted effect estimates	3	1.06 (0.71-1.56)	0.785	55.0	0.108
Published estimates only	3	1.76 (1.34-2.32)	<0.001	0.0	0.553
Unpublished estimates only	9	1.00 (0.73-1.38)	0.980	33.0	0.154
Only studies with low/medium risk of bias	6	1.06 (0.66-1.71)	0.802	69.0	0.007
Only longitudinal studies	10	1.15 (0.81-1.62)	0.436	55.1	0.018
Only hazard ratios	6	1.05 (0.67-1.64)	0.842	69.1	0.006
Excluding studies with zero cases	10	1.27 (0.95-1.70)	0.110	58.2	0.010
Only studies with >90% injecting in last 6 months	5	0.96 (0.63-1.48)	0.864	58.2	0.048
**Recent incarceration on HCV acquisition risk**					
Crude effect estimates	17	1.62 (1.28-2.05)	<0.001	57.3	0.002
Adjusted effect estimates	11	1.60 (1.21-2.11)	0.001	64.7	0.002
Published estimates only	7	1.58 (1.02-2.45)	0.041	68.4	0.004
Unpublished estimates only	10	1.61 (1.22-2.12)	0.001	45.6	0.057
Only studies with low/medium risk of bias	7	1.70 (1.20-2.43)	0.003	67.0	0.006
Only longitudinal studies	14	1.59 (1.22-2.08)	0.001	64.2	0.001
Only hazard ratios	7	1.65 (1.15-2.37)	0.006	79.1	<0.001
Only studies with >90% injecting in last 6 months	7	1.55 (1.11-2.15)	0.009	61.4	0.016
**Past incarceration on HCV acquisition risk**					
Crude effect estimates	22	1.21 (1.02-1.43)	0.027	50.6	0.004
Adjusted effect estimates	6	1.12 (0.88-1.42)	0.366	57.5	0.038
Published estimates only	11	1.39 (1.11-1.74)	0.004	46.3	0.046
Unpublished estimates only	10	1.05 (0.84-1.30)	0.680	35.5	0.115
Only studies with low/medium risk of bias	7	0.96 (0.75-1.22)	0.724	25.2	0.236
Only longitudinal studies	18	1.18 (0.97-1.44)	0.102	56.0	0.002
Only hazard ratios	4	0.92 (0.68-1.25)	0.595	24.1	0.267
Only studies with >90% injecting in last 6 months	8	1.03 (0.80-1.32)	0.824	39.6	0.115

Appendix Table 12: Uni-variable meta-regression for the effect of recent incarceration on the risk of HIV acquisition.

	Number of studies	Effect size (95% CI)	Ratio (95% CI)	τ^2	Adjusted R^2	P-value
**Region**				0.052	54.72	0.1670
North America	4	1.56 (1.16-2.11)	1.00			
East and South-East Asia	3	2.69 (1.86-3.90)	1.72 (1.01-2.94)			
Eastern Europe	2	2.34 (0.72-7.60)	1.50 (0.34-6.57)			
Latin America	2	0.68 (0.30-1.57)	0.44 (0.14-1.38)			
South Asia	2	1.36 (0.45-4.11)	0.98 (0.37-2.60)			
Australasia	1	2.16 (0.70-6.65)	1.39 (0.32-5.99)			
**Economic level**				0.087	24.51	0.303
High income country	5	1.58 (1.20-2.09)	1.00			
Lower/upper middle-income country	9	2.01 (1.37-2.94)	1.30 (0.77-2.19)			
**Females (%)**				0.097	20.06	0.143
10% increase	13		0.84 (0.65-1.07)			
**Age (mean/median)**				0.052	60.62	0.016
<34.3 years	6	2.40 (1.87-3.09)	1.00			
>=34.3 years	6	1.28 (0.91-1.81)	0.54 (0.33-0.87)			
**Duration of injecting (mean/median)**				0.073	36.41	0.137
<13.8 years	4	2.49 (1.64-3.77)	1.00			
>=13.8 years	4	1.29 (0.83-1.99)	0.52 (0.27-1.00)			
Not reported	6	1.89 (1.36-2.62)	0.76 (0.41-1.40)			
**Baseline HIV prevalence**				0.124	-7.57	0.346
Per 10 % increase	14		1.13 (0.86-1.49)			
**Proportion homeless**				0.179	-55.60	0.405
<25.2%	3	1.20 (0.54-2.66)	1.00			
>=25.2%	3	1.77 (1.34-2.34)	1.27 (0.45-3.61)			
Not reported	8		1.71 (0.70-4.17)			
**OST coverage**				0.064	43.25	0.152
<19.4%	3	2.13 (1.00,4.52)	1.00			
>=19.4%	3	2.04 (1.60-2.60)	0.84 (0.42-1.68)			
Not reported	8	1.43 (1.05-1.95)	0.57 (0.30-1.10)			
**Study design**				0.123	-7.27	0.801
Longitudinal	12	1.79 (1.36-2.36)	1.00			
Cross-sectional	2	2.05 (0.98-4.27)	1.14 (0.38-3.38)			
**Effect type**				0.159	-38.19	0.987
Hazard ratio	7	1.87 (1.52-2.31)	1.00			
Incidence rate ratio	5	1.59 (0.70-3.57)	0.92 (0.45-1.90)			
Odds ratio	1	1.91 (0.26-14.18)	1.04 (0.09-12.48)			
Risk ratio	1	2.07 (0.94-4.56)	1.13 (0.30-4.28)			
**Start of study**				0.130	-12.67	0.557
<2001	7	1.92 (1.39-2.65)	1.00			
>=2001	7	1.60 (1.00-2.56)	0.84 (0.45-1.57)			
**Midpoint of study**				0.141	-22.53	0.866
<2005.5	7	1.84 (1.23-2.75)	1.00			
>=2005.5	7	1.80 (1.32-2.46)	0.95 (0.52-1.67)			
**Length of study**				0.077	33.32	0.104
<5 years	6	2.46 (1.92-3.15)	1.00			
>=5 years	8	1.57 (1.16-2.13)	0.65 (0.38-1.11)			
**Prison population rate**				0.144	-24.78	0.793
Low (< global average - 144 per 100,000)	5	1.8 (1.47-2.20)	1.00			
High (>= global average - 144 per 100,000)	9	1.85 (1.19-2.87)	1.08 (0.58-2.01)			
**Recent incarceration definition**				0.046	59.88	0.081
Last 6 months	10	1.55 (1.22-1.95)	1.00			
Last 12 months	2	2.32 (0.95-5.64)	1.50 (0.50-4.50)			
Last 3 months	1	3.10 (1.71-5.63)	2.00 (0.84-4.77)			
Since last visit	1	3.39 (2.35-4.90)	2.19 (1.10-4.35)			
Per 3-month increase	13		0.95 (0.60-1.51)	0.068	-9.23	0.819
**Publication status**						
Unpublished	10	1.47 (1.14-1.90)	1.00	0.046	59.67%	0.018
Published	4	2.59 (1.91-3.52)	1.76 (1.12-2.76)			

Appendix Table 13: Uni-variable meta-regression for the effect of past incarceration on the risk of HIV acquisition.

	Number of studies	Effect size (95% CI)	Ratio (95% CI)	τ^2	Adjusted R^2	P-value
**Region**				0.046	53.06	0.306
East and South-East Asia	3	1.62 (1.20-2.20)	1.00			
Eastern Europe	2	1.48 (0.42-4.94)	0.90 (0.26-3.10)			
Latin America	2	0.60 (0.32-1.13)	0.37 (0.13-1.09)			
North America	2	1.13 (0.60-2.13)	0.69 (0.3-1.47)			
Australasia	1	0.71 (0.24-2.12)	0.44 (0.09-2.25)			
South Asia	1	1.44 (0.42-4.94)	0.89 (0.15-5.38)			
Western Europe	1	2.40 (1.27-4.53)	1.49 (0.47-4.66)			
**Economic level**				0.123	-26.06	0.947
High income country	5	1.25 (0.76-2.05)	1.00			
Lower/upper middle-income country	7	1.27 (0.89-1.79)	0.98 (0.50-1.93)			
**Females (%)**				0.012	87.53	0.020
10% increase	11		0.78 (0.64-0.95)			
**Age (mean/median)**				0.064	48.42	0.126
<35.4 years	5	1.60 (1.23-2.08)	1.00			
>=35.4 years	5	0.96 (0.54-1.71)	0.62 (0.32-1.18)			
**Duration of injecting (mean/median)**				0.040	58.93	0.121
<12.8 years	4	1.71 (1.32-2.23)	1.00			
>=12.8 years	4	0.92 (0.69-1.22)	0.56 (0.30-1.02)			
Not reported	4	0.94 (0.45-1.97)	0.63 (0.32-1.21)			
**Baseline HIV prevalence**				0.127	-29.31	0.323
Per 10 % increase	12		1.15 (0.85-1.57)			
**Proportion homeless**				0.065	33.11	0.152
<23.1%	3	0.61 (0.33-1.12)	1.00			
>=23.1%	4	1.39 (0.83-2.34)	2.19 (0.87-5.49)			
Not reported	5	1.53 (1.17-2.02)	2.39 (0.94-6.06)			
**OST coverage**				0.027	72.40	0.077
<19.4%	3	1.86 (1.31-2.64)	1.00			
>=19.4%	2	0.82 (0.58-1.15)	0.44 (0.21-0.89)			
Not reported	7	1.17 (0.82-1.67)	0.66 (0.37-1.18)			
**Study design**				0.109	-11.00	0.375
Longitudinal	10	1.15 (0.81-1.62)	1.00			
Cross-sectional	2	1.60 (1.10-2.31)	1.39 (0.63-3.06)			
**Effect type**				0.149	-52.06	0.735
Hazard ratio	6	1.05 (0.67-1.64)	1.00			
Incidence rate ratio	4	1.59 (1.06-2.40)	1.36 (0.52-3.54)			
Odds ratio	1	1.65 (0.76-3.59)	1.56 (0.40-6.11)			
Risk ratio	1	1.65 (1.09-2.51)	1.50 (0.48-4.62)			
**Start of study**				0.122	-20.09	0.909
<2002	6	1.27 (0.90-1.79)	1.00			
>=2002	6	1.19 (0.68-2.09)	0.96 (0.48-1.94)			
**Midpoint of study**				0.118	-20.33	0.645
<2005.5	5	1.32 (0.93-1.88)	1.00			
>=2005.5	7	1.12 (0.68-1.85)	0.87 (0.44-1.70)			
**Length of study**				0.021	78.06	0.019
<4 years	5	1.74 (1.34-2.25)	1.00			
>=4 years	7	0.93 (0.64-1.35)	0.56 (0.35-0.89)			
**Prison population rate**				0.129	-32.00	0.549
Low (< global average - 144 per 100,000)	4	1.39 (0.83-2.34)	1.00			
High (>= global average - 144 per 100,000)	8	1.18 (0.83-1.70)	0.82 (0.41-1.65)			
**Past incarceration definition**				0.090	10.11	0.344
Baseline	9	1.07 (0.78-1.47)	1.00			
Measured at first follow-up	1	1.44 (0.42-4.94)	1.35 (0.26-6.99)			
Time varying	1	2.40 (1.27-4.53)	2.24 (0.76-6.61)			

	Number of studies	Effect size (95% CI)	Ratio (95% CI)	τ^2	Adjusted R^2	P-value
Time varying measured at preceding visit	1	1.70 (1.09-2.65)	1.59 (0.62-4.06)			
**Publication status**				0.034	64.98	0.034
Unpublished	9	1.00 (0.73-1.38)	1.00			
Published	3	1.76 (1.34-2.32)	1.76 (1.05-2.92)			

Appendix Table 14: Multi-variable meta-regression for the effect of recent incarceration on the risk of HIV acquisition.

	Ratio (95% CI)	P-Value
**Age (mean/median)**		
<33.6 years	1.00	
>=33.6 years	0.59 (0.17-2.09)	0.332
**Proportion homeless**		
<25.2%	1.00	
>=25.2%	1.17 (0.26-5.35)	0.801
Not reported	1.12 (0.23-5.58)	0.860
**OST coverage**		
<19.4%	1.00	
>=19.4%	1.05 (0.29-3.80)	0.929
Not reported	0.75 (0.14-4.02)	0.682
**Publication status**		
Unpublished	1.00	
Published	0.97 (0.11-8.56)	0.566

Appendix Table 15: Multi-variable meta-regression for the effect of past incarceration on the risk of HIV acquisition.

	Ratio (95% CI)	P-Value
**Females (%)**		
10% increase	0.97 (0.55-1.74)	0.896
**Proportion homeless**		
<23.1%	1.00	
>=23.1%	2.44 (0.73-8.22)	0.101
Not reported	1.53 (0.25-9.30)	0.505
**OST coverage**		
<19.4%	1.00	
>=19.4%	0.56 (0.11-2.71)	0.323
Not reported	0.99 (0.39-2.51)	0.970
**Length of study**		
<4 years	1.00	
>=4 years	0.57 (0.10-3.35)	0.383
**Publication status**		
Unpublished	1.00	
Published	0.93 (0.20-4.23)	0.888

Appendix Table 16: Uni-variable meta-regression for the effect of recent incarceration on the risk of HCV acquisition.

	Number of studies	Effect size (95% CI)	Ratio (95% CI)	τ^2	Adjusted R^2	P-value
**Region**				0.080	31.40	0.360
North America	8	1.59 (1.17-2.16)	1.00			
Western Europe	4	1.18 (0.77-1.82)	0.77 (0.41-1.45)			
Australasia	3	2.78 (2.01-3.83)	1.71 (0.86-3.38)			
Eastern Europe	1	1.56 (0.57-4.23)	0.99 (0.26-3.65)			
South Asia	1	1.36 (0.60-3.09)	0.86 (0.27-2.71)			
**Economic level**				0.125	-7.44	0.772
High income country	15	1.64 (1.27-2.12)	1.00			
Lower/upper middle-income country	2	1.44 (0.76-2.71)	0.88 (0.35-2.21)			
**Females (%)**				0.141	-13.60	0.738
10% increase	16		1.03 (0.84-1.27)			
**Age (mean/median)**				0.094	-34.27	0.695
<29.8 years	7	1.40 (0.99-1.97)	1.00			
>=29.8 years	6	1.55 (1.06-2.27)	1.11 (0.62-1.99)			
**Duration of injecting (mean/median)**				0.102	-1.77	0.344
<7 years	5	1.43 (0.85-2.39)	1.00			
>=7 years	5	2.00 (1.36-2.92)	1.38 (0.66-2.91)			
**Baseline HCV prevalence**				0.091	17.65	0.257
Per 10 % increase	12		0.92 (0.79-1.07)			
**Proportion homeless**				.116	1.09	0.610
<42.8%	4	1.27 (0.99-1.63)	1.00			
>=42.8%	5	1.67 (1.06-2.64)	1.25 (0.65-2.39)			
Not reported	8	1.78 (1.15-2.74)	1.34 (0.71-2.55)			
**OST coverage**				0.091	7.47%	0.447
<33.1%	5	1.66 (1.12-2.44)	1.00			
>=33.1%	5	2.30 (1.70-3.12)	1.26 (0.65-2.42)			
**Study design**				0.125	-7.39	0.758
Longitudinal	14	1.59 (1.22-2.08)	1.00			
Cross-sectional	3	1.79 (1.06-3.02)	1.12 (0.52-2.42)			
**Effect type**				0.136	-16.50	0.885
Hazard ratio	7	1.65 (1.15-2.37)	1.00			
Incidence rate ratio	7	1.46 (1.00-2.12)	0.90 (0.49-1.64)			
Risk ratio	3	1.79 (1.06-3.02)	1.08 (0.48-2.44)			
**Start of study**				0.139	-19.35%	0.642
<2003	6	1.50 (0.95-2.35)	1.00			
>=2003	11	1.69 (1.27-2.26)	1.13 (0.65-1.96)			
**Midpoint of study**				0.123	-5.24	0.951
<2006	7	1.57 (0.98-2.53)	1.00			
>=2006	10	1.62 (1.23-2.13)	1.02 (0.59-1.76)			
**Length of study**				0.129	-10.68	0.740
<7 years	8	1.45 (1.10-1.90)	1.00			
>=7 years	9	1.66 (1.17-2.37)	1.09 (0.63-1.89)			
**Prison population rate**				0.046	60.50	0.022
Low (< global average - 144 per 100,000)	11	1.40 (1.10-1.80)	1.00			
High (>= global average - 144 per 100,000)	6	2.57 (1.95-3.38)	1.74 (1.09-2.76)			
**Recent incarceration definition**				0.080	31.42	0.404
Last 6 months	8	1.54 (1.16-2.05)	1.00			
Last 12 months	5	2.15 (1.50-3.07)	1.31 (0.74-2.31)			
Last 3 months	2	1.75 (0.74-4.12)	1.22 (0.56-2.68)			
Last 2 years	1	2.75 (0.34-21.99)	1.79 (0.16-19.92)			
Since last visit	1	0.79 (0.42-1.48)	0.51 (0.19-1.37)			
Per 3-month increase	16		1.08 (0.87-1.34)	0.09	3.10	0.445
**Publication status**				0.124	57.78%	0.961
Unpublished	10	1.61 (1.22-2.12)	1.00			
Published	7	1.58 (1.02-2.45)	0.99 (0.58-1.70)			

Appendix Table 17: Uni-variable meta-regression for the effect of past incarceration on the risk of HCV acquisition.

	Number of studies	Effect size (95% CI)	Ratio (95% CI)	τ^2	Adjusted R^2	P-value
**Region**				0.072	-7.10	0.404
North America	7	1.03 (0.86-1.24)	1.00			
Western Europe	7	1.31 (1.14-1.51)	1.28 (0.84-1.96)			
Australasia	5	0.99 (0.35-2.86)	1.05 (0.62-1.78)			
Eastern Europe	2	0.67 (0.28-1.60)	0.61 (0.21-1.77)			
South Asia	1	2.04 (0.9-4.65)	1.87 (0.63-5.58)			
**Economic level**				0.071	-5.68	0.808
High income country	20	1.21 (1.02-1.43)	1.00			
Lower/upper middle-income country	2	1.13 (0.29-4.44)	1.11 (0.46-2.71)			
**Females (%)**				0.074	-4.44	0.482
10% increase	21		1.05 (0.91-1.21)			
**Age (mean/ median)**				0.065	9.41	0.377
<25 years	10	1.29 (0.94-1.76)	1.00			
>=25 years	11	1.12 (0.92-1.37)	0.85 (0.58-1.24)			
**Duration of injecting (mean/ median)**				0.063	-19.80	0.667
<7 years	7	1.05 (0.75-1.47)	1.00			
>=7 years	8	1.15 (0.91-1.45)	1.10 (0.70-1.73)			
**Baseline HCV prevalence**				0.020	12.57	0.237
Per 10 % increase	16		0.94 (0.86-1.04)			
**Proportion homeless**				0.044	-15.68	0.469
<29.7%	7	1.22 (0.89-1.67)	1.00			
>=29.7%	8	1.03 (0.82-1.29)	0.87 (0.57-1.32)			
**OST coverage**				0.027	-21.72	0.743
<31.0%	7	1.04 (0.83-1.30)	1.00			
>=31.0%	8	1.12 (0.86-1.46)	1.06 (0.72-1.56)			
**Study design**				0.077	-14.78	0.561
Longitudinal	19	1.18 (0.97-1.44)	1.00			
Cross-sectional	3	1.27 (0.98-1.66)	1.15 (0.70-1.92)			
**Effect type**				0.070	-4.50	0.4497
Incidence rate ratio	14	1.25 (0.95-1.64)	1.00			
Hazard ratio	4	0.92 (0.68-1.25)	0.73 (0.45-1.18)			
Risk ratio	3	1.25 (1.07-1.46)	0.99 (0.62-1.57)			
Odds ratio	1	1.74 (0.93-3.26)	1.36 (0.55-3.38)			
**Start of study**				0.074	-10.95	0.411
<2002	9	1.31 (0.97-1.78)	1.00			
>=2002	13	1.13 0.94-1.36)	0.86 (0.59-1.25)			
**Midpoint of study**				0.040	39.95	0.024
<2005.8	11	1.48 (1.18-1.86)	1.00			
>=2005.8	11	1.02 (0.84-1.24)	0.69 (0.50-0.95)			
**Length of study**				0.076	-14.39	0.766
<5 years	8	1.18 (0.90-1.55)	1.00			
>=5 years	14	1.23 (0.99-1.53)	1.06 (0.70-1.60)			
**Prison population rate**				0.077	-14.55	0.497
Low (< global average - 144 per 100,000)	12	1.22 (1.05-1.43)	1.00			
High (>= global average - 144 per 100,000)	10	1.03 (0.69-1.54)	0.88 (0.60-1.29)			
**Past incarceration definition**				0.077	-7.11	0.591
Baseline	19	1.19 (0.99-1.44)	1.00			
Measured at first follow-up	1	2.04 (0.90-4.65)	1.71 (0.58-5.10)			
Measured during last period of follow up	1	1.20 (0.57-2.50)	1.00 (0.36-2.76)			
**Publication status**				0.045	20.80	0.177
Unpublished	11	1.10 (0.88-1.38)	1.00			
Published	11	1.39 (1.11-1.74)	1.26 (0.89-1.77)			

Appendix Table 18: Multi-variable meta-regression for the effect of recent incarceration on the risk of HCV acquisition.

	Ratio (95% CI)	P-Value
**Proportion homeless**		
<42.8%	1.00	
>=42.8%	1.62 (0.88-2.99)	0.110
Not reported	0.95 (0.35-2.54)	0.907
**OST coverage**		
<33.1%	1.00	
>=33.1%	0.99 (0.51-1.70)	0.979
Not reported	0.66 (0.36-1.23)	0.405
**Prison Population Rate**		
Low (< Global Average - 144 per 100,000)	1.00	
High (>= Global Average - 144 per 100,000)	2.25 (0.91-1.91)	0.077

Appendix Table 19: Multi-variable meta-regression for the effect of past incarceration on the risk of HCV acquisition.

	Ratio (95% CI)	P-Value
**Proportion homeless**		
<29.7%	1.00	
>=29.7%	0.73 (0.41-1.29)	0.242
**OST coverage**		
<31.0%	1.00	
>=31.0%	0.91 (0.51-1.62)	0.714
**Midpoint of Study**		
<2005.8	1.00	
>=2005.8	0.73 (0.42-1.26)	0.221

Appendix Table 20: Analysis of confounding: uni-variable meta-regression for the adjusted effect of recent incarceration on the risk of HCV acquisition.

	Number of studies	Effect size (95% CI)	Ratio (95% CI)	τ^2	Adjusted R^2	P-value
**Adjusted for recent homelessness**						
No	4	2.27 (1.71-3.03)	1.00			
Yes	7	1.40 (1.03-1.90)	0.66 (0.37-1.17)	0.068	44.00	0.132
**Adjusted for recent OST exposure**						
No	3	2.16 (1.44-3.24)	1.00			
yes	8	1.44 (1.08-1.92)	0.68 (0.37-1.26)	0.075	38.01	0.192
**Adjusted for recent stimulant injecting**						
No	2	1.64 (1.18-2.26)	1.00			
yes	9	1.56 (1.11-2.21)	0.90 (0.37-2.20)	0.156	-29.12	0.795

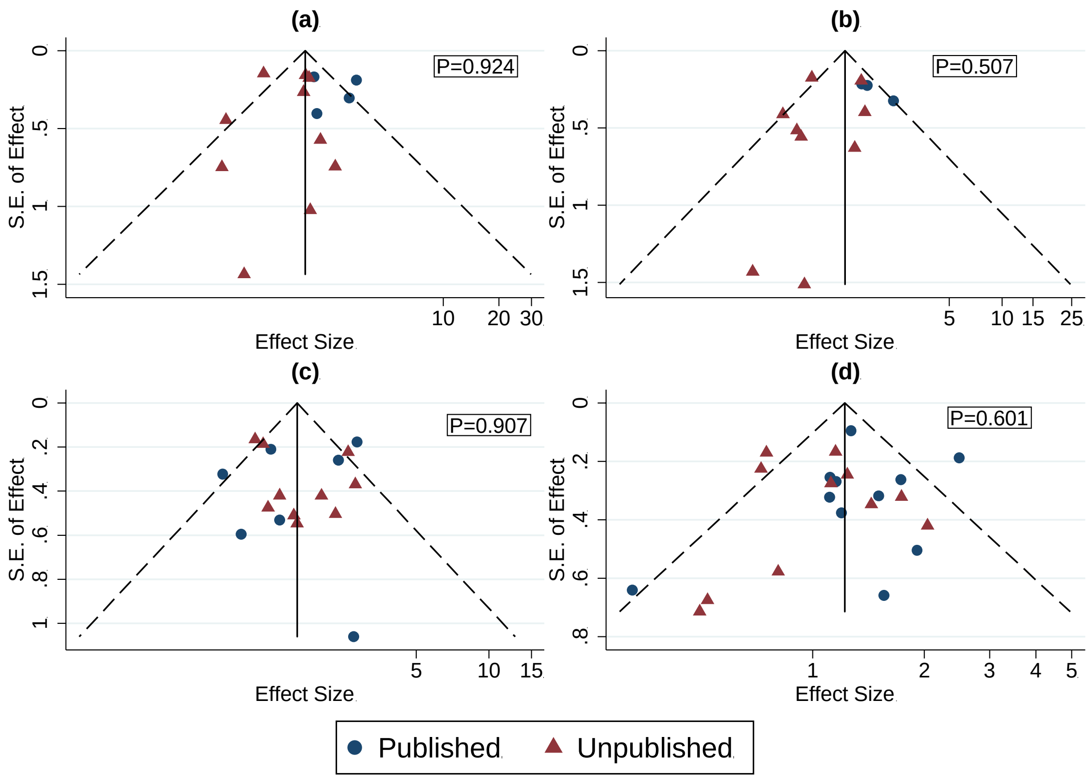

Appendix Figure 1: Funnel plot of studies included in the meta-analysis of: (a) the effect of recent incarceration on HIV acquisition risk; (b) the effect of past incarceration on HIV acquisition risk; (c) the effect of recent incarceration on HCV acquisition risk; (d) the effect of past incarceration on HCV acquisition risk. The central lines are plotted at the fixed-effect summary effects while boxes show the p-values for Egger's tests.

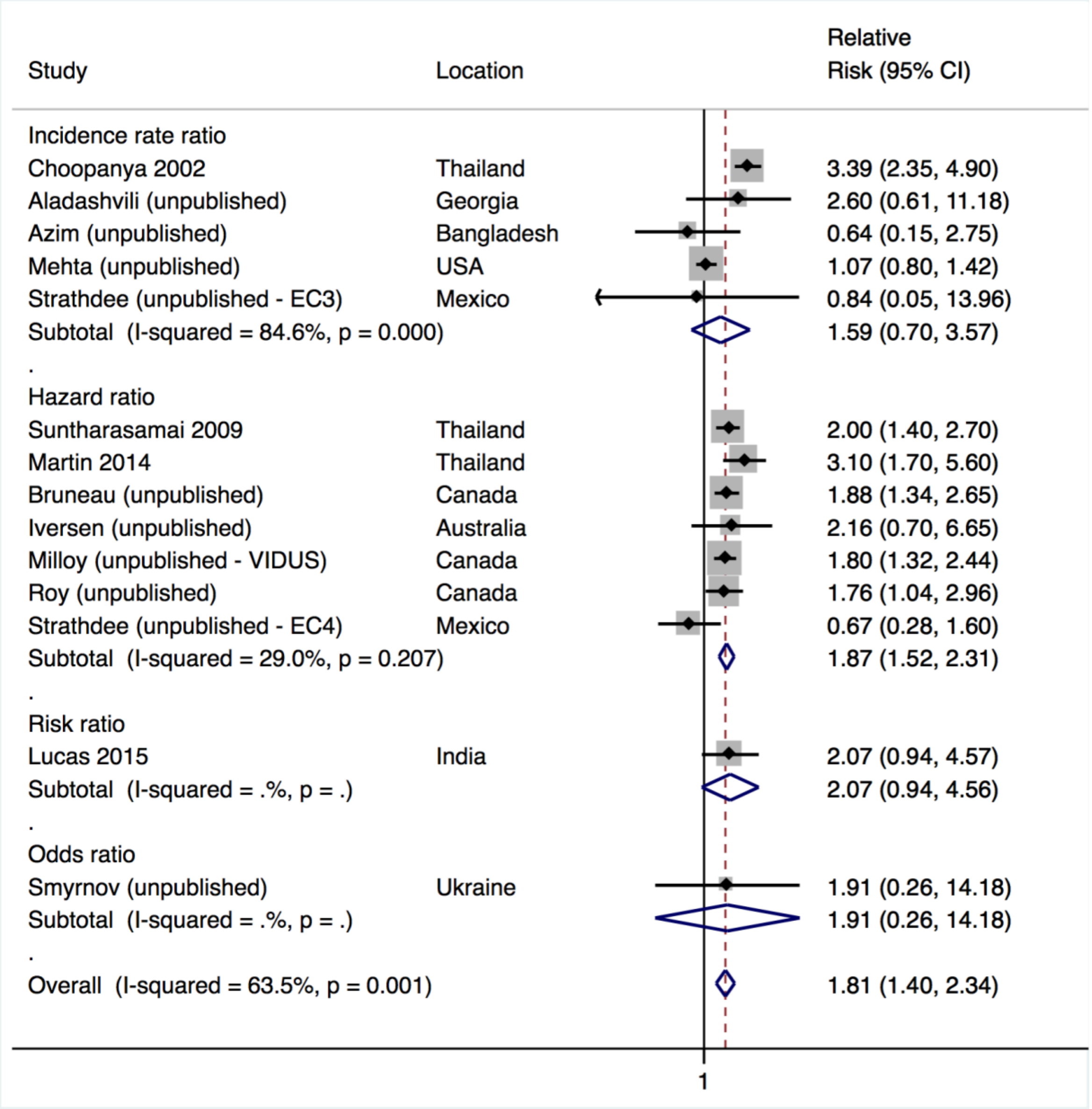

Appendix Figure 2: Meta-analysis of studies showing the crude effect of recent incarceration on the risk of HIV acquisition among PWID, by effect type.

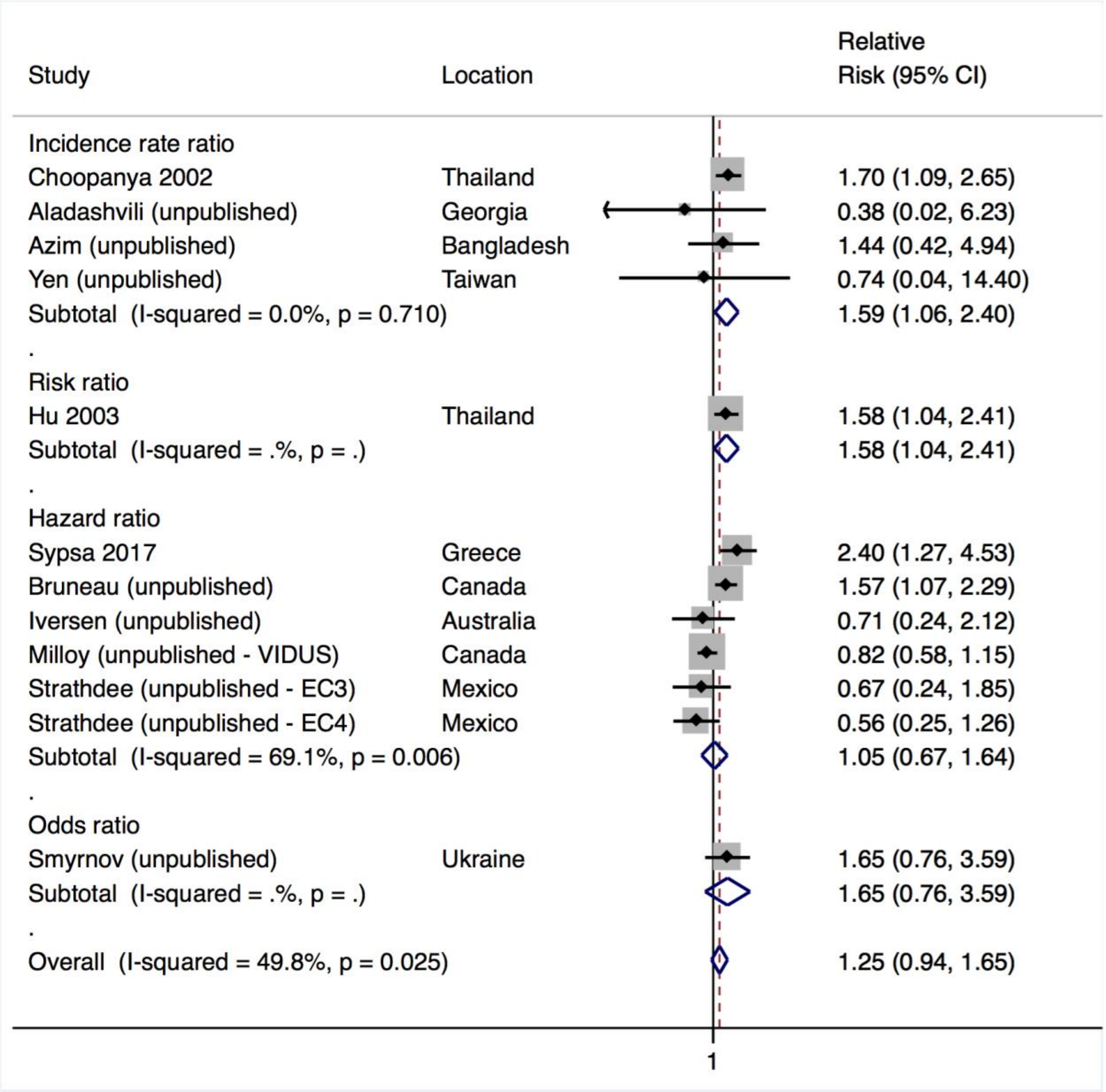

Appendix Figure 3: Meta-analysis of studies showing the crude effect of past incarceration on the risk of HIV acquisition among PWID, by effect type.

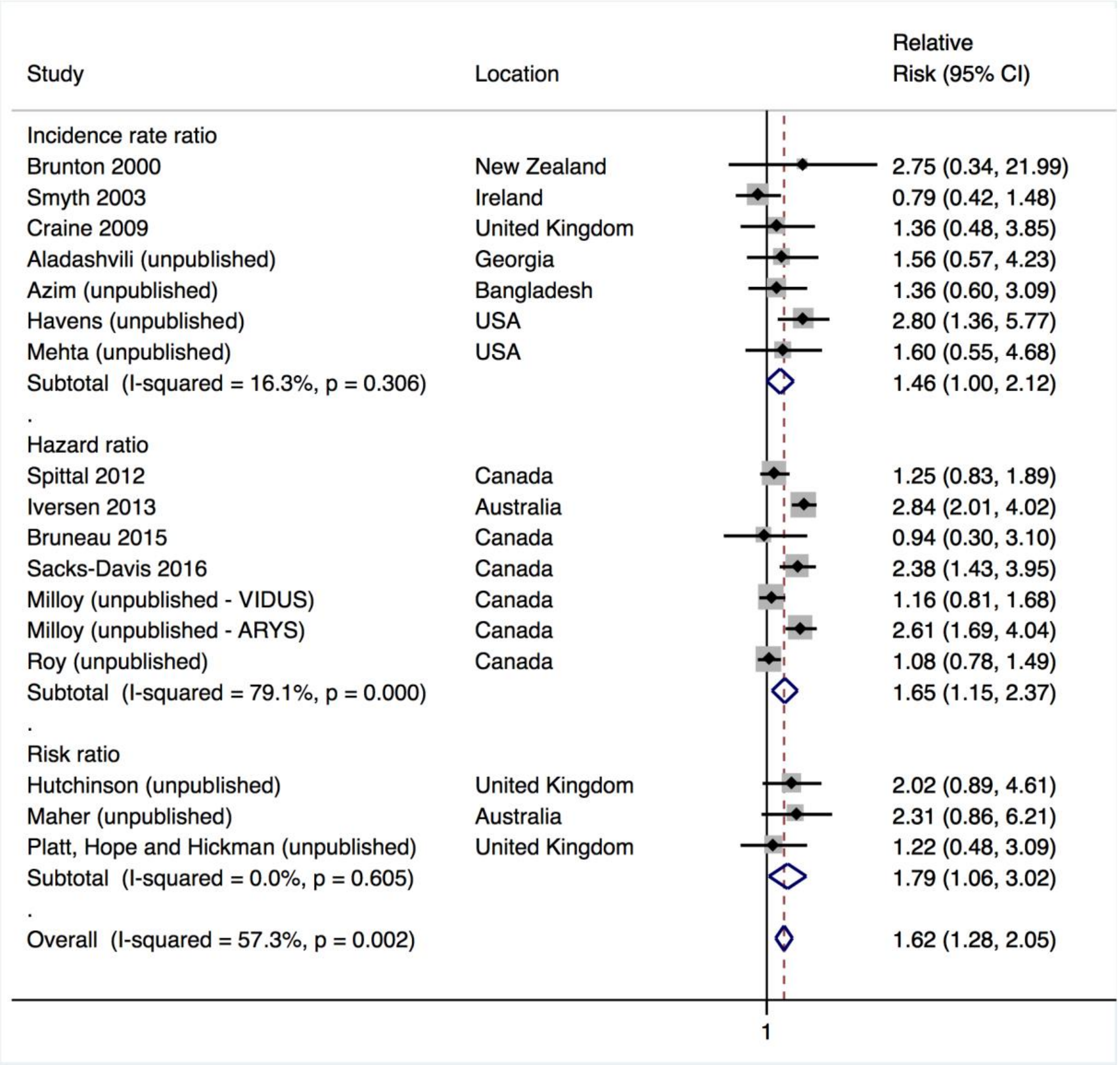

Appendix Figure 4: Meta-analysis of studies showing the crude effect of recent incarceration on the risk of HCV acquisition among PWID, by effect type.

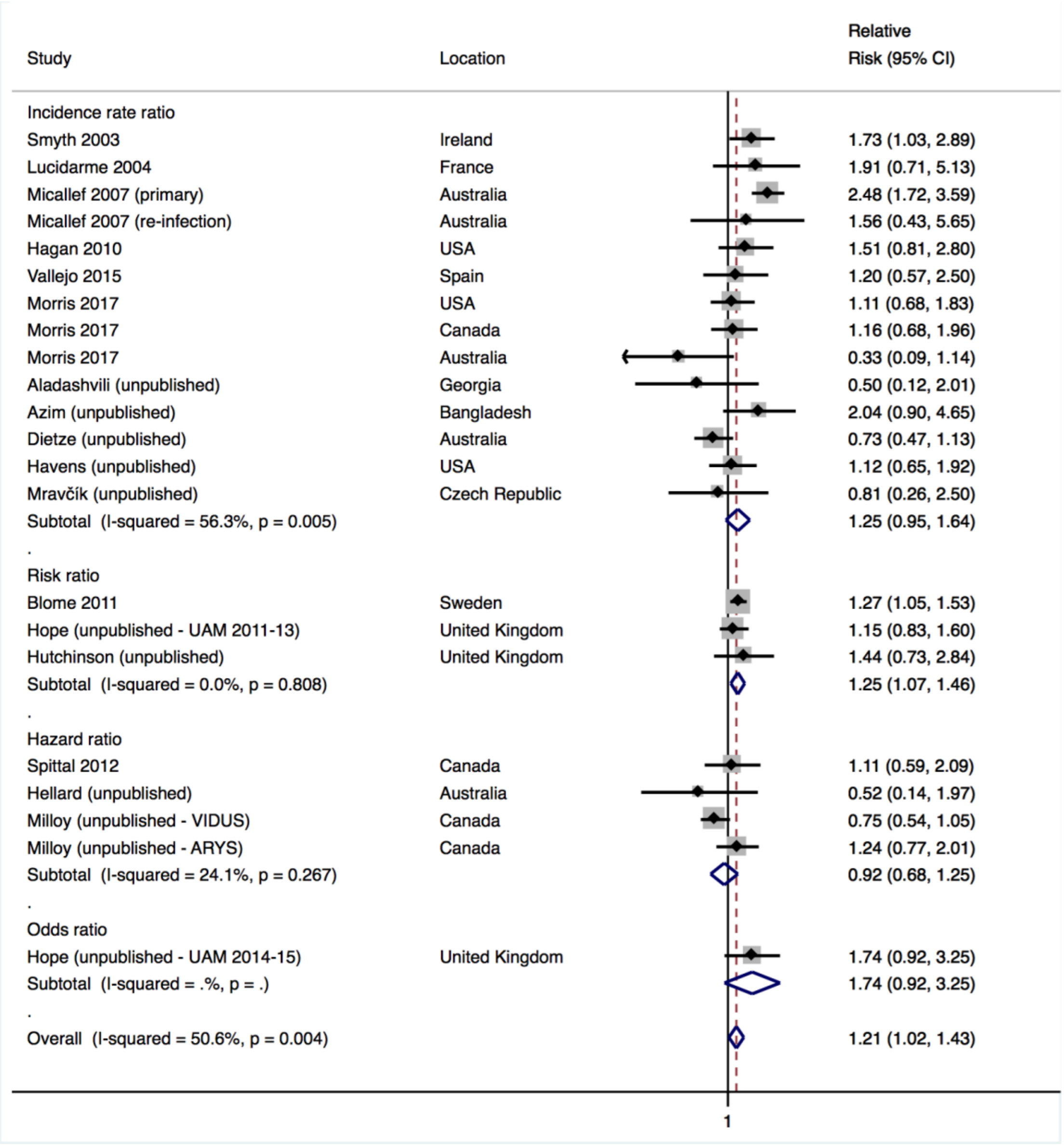

Appendix Figure 5: Meta-analysis of studies showing the crude effect of past incarceration on the risk of HCV acquisition among PWID, by effect type.

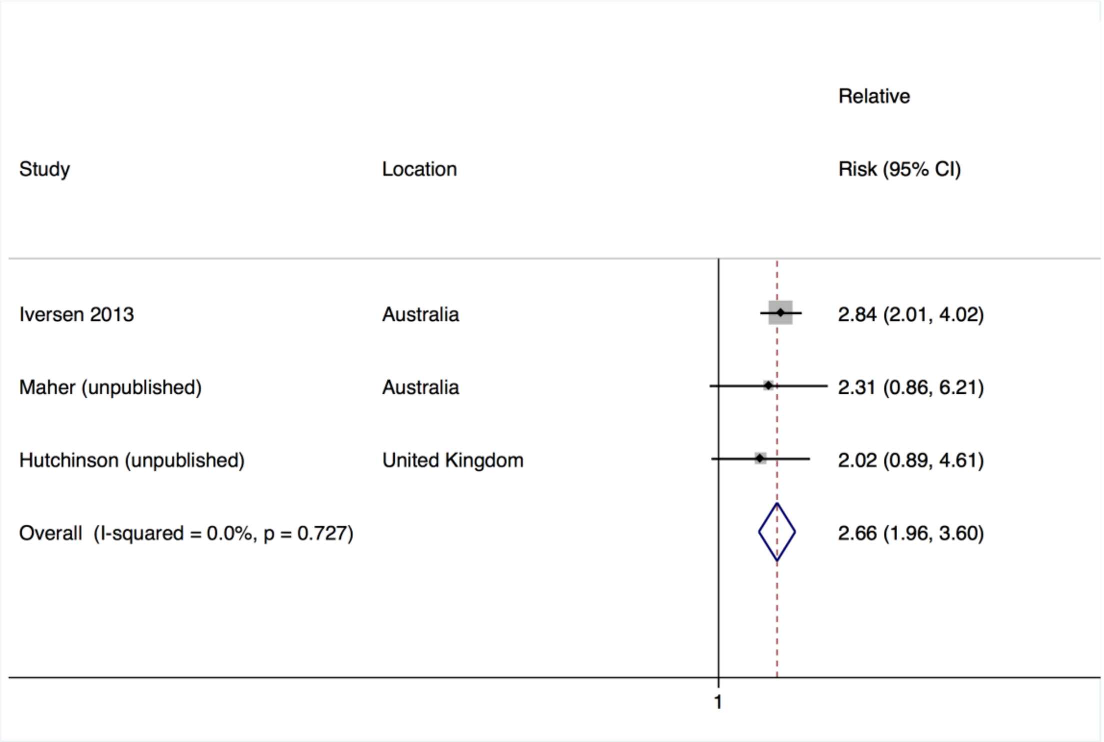

Appendix Figure 6: Meta-analysis of the crude effect of recent incarceration on the risk of HCV acquisition among PWID in settings with low HCV incidence in prisons showing.

References

1. Ahamad K, Hayashi K, Nguyen P, et al. Effect of low-threshold methadone maintenance therapy for people who inject drugs on HIV incidence in Vancouver, BC, Canada: an observational cohort study. *Lancet HIV* 2015; **2**(10): e445-50.
2. Aitken CK, Lewis J, Tracy SL, et al. High incidence of hepatitis C virus reinfection in a cohort of injecting drug users. *Hepatology* 2008; **48**(6): 1746-52.
3. Allen EJ, Palmateer NE, Hutchinson SJ, Cameron S, Goldberg DJ, Taylor A. Association between harm reduction intervention uptake and recent hepatitis C infection among people who inject drugs attending sites that provide sterile injecting equipment in Scotland. *Int J Drug Policy* 2012; **23**(5): 346-52.
4. Boileau C, Bruneau J, Al-Nachawati H, Lamothe F, Vincelette J. A prognostic model for HIV seroconversion among injection drug users as a tool for stratification in clinical trials. *J Acquir Immune Defic Syndr* 2005; **39**(4): 489-95.
5. Bruneau J, Roy E, Arruda N, Zang G, Jutras-Aswad D. The rising prevalence of prescription opioid injection and its association with hepatitis C incidence among street-drug users. *Addiction* 2012; **107**(7): 1318-27.
6. Craib KJ, Spittal PM, Wood E, et al. Risk factors for elevated HIV incidence among Aboriginal injection drug users in Vancouver. *Cmaj* 2003; **168**(1): 19-24.
7. Cullen KJ, Hope VD, Croxford S, Shute J, Ncube F, Parry JV. Factors associated with recently acquired hepatitis C virus infection in people who inject drugs in England, Wales and Northern Ireland: new findings from an unlinked anonymous monitoring survey. *Epidemiol Infect* 2015; **143**(7): 1398-407.
8. Hahn JA, Page-Shafer K, Lum PJ, et al. Hepatitis C virus seroconversion among young injection drug users: relationships and risks. *J Infect Dis* 2002; **186**(11): 1558-64.
9. Hope VD, Hickman M, Ngui SL, et al. Measuring the incidence, prevalence and genetic relatedness of hepatitis C infections among a community recruited sample of injecting drug users, using dried blood spots. *J Viral Hepat* 2011; **18**(4): 262-70.
10. Hudgens MG, Longini IM, Jr., Vanichseni S, et al. Subtype-specific transmission probabilities for human immunodeficiency virus type 1 among injecting drug users in Bangkok, Thailand. *Am J Epidemiol* 2002; **155**(2): 159-68.
11. Kerr T, Shannon K, Ti L, et al. Sex work and HIV incidence among people who inject drugs. *AIDS* 2016; **30**(4): 627-34.
12. Martin M, Vanichseni S, Suntharasamai P, et al. Drug use and the risk of HIV infection amongst injection drug users participating in an HIV vaccine trial in Bangkok, 1999-2003. *Int J Drug Policy* 2010; **21**(4): 296-301.
13. Montain J, Ti L, Hayashi K, Nguyen P, Wood E, Kerr T. Impact of length of injecting career on HIV incidence among people who inject drugs. *Addict Behav* 2016; **58**: 90-4.
14. Morris MD, Shiboski S, Bruneau J, et al. Geographic Differences in Temporal Incidence Trends of Hepatitis C Virus Infection Among People Who Inject Drugs: The InC3 Collaboration. *Clin Infect Dis* 2017; **64**(7): 860-9.
15. Nelson KE, Galai N, Safaeian M, Strathdee SA, Celentano DD, Vlahov D. Temporal trends in the incidence of human immunodeficiency virus infection and risk behavior among injection drug users in Baltimore, Maryland, 1988-1998. *Am J Epidemiol* 2002; **156**(7): 641-53.
16. Patrick DM, Tyndall MW, Cornelisse PG, et al. Incidence of hepatitis C virus infection among injection drug users during an outbreak of HIV infection. *Cmaj* 2001; **165**(7): 889-95.
17. Spittal PM, Craib KJ, Wood E, et al. Risk factors for elevated HIV incidence rates among female injection drug users in Vancouver. *Cmaj* 2002; **166**(7): 894-9.
18. Stone J, Martin NK, Hickman M, et al. Modelling the impact of incarceration and prison-based hepatitis C virus (HCV) treatment on HCV transmission among people who inject drugs in Scotland. *Addiction* 2017; **112**(7): 1302-14.
19. Strathdee SA, Galai N, Safaiean M, et al. Sex differences in risk factors for hiv seroconversion among injection drug users: a 10-year perspective. *Arch Intern Med* 2001; **161**(10): 1281-8.
20. Tyndall MW, Currie S, Spittal P, et al. Intensive injection cocaine use as the primary risk factor in the Vancouver HIV-1 epidemic. *AIDS* 2003; **17**(6): 887-93.
21. Vanichseni S, Kitayaporn D, Mastro TD, et al. Continued high HIV-1 incidence in a vaccine trial preparatory cohort of injection drug users in Bangkok, Thailand. *AIDS* 2001; **15**(3): 397-405.
22. Choopanya K, Des Jarlais DC, Vanichseni S, et al. Incarceration and risk for HIV infection among injection drug users in Bangkok. *J Acquir Immune Defic Syndr* 2002; **29**(1): 86-94.
23. Lucas GM, Solomon SS, Srikrishnan AK, et al. High HIV burden among people who inject drugs in 15 Indian cities. *AIDS* 2015; **29**(5): 619-28.
24. Martin M, Vanichseni S, Suntharasamai P, et al. Risk behaviors and risk factors for HIV infection among participants in the Bangkok tenofovir study, an HIV pre-exposure prophylaxis trial among people who inject drugs. *PLoS One* 2014; **9**(3): e92809.

25. Suntharasamai P, Martin M, Vanichseni S, et al. Factors associated with incarceration and incident human immunodeficiency virus (HIV) infection among injection drug users participating in an HIV vaccine trial in Bangkok, Thailand, 1999-2003. *Addiction* 2009; **104**(2): 235-42.
26. Hu DJ, Vanichseni S, Mock PA, et al. HIV type 1 incidence estimates by detection of recent infection from a cross-sectional sampling of injection drug users in Bangkok: use of the IgG capture BED enzyme immunoassay. *AIDS Res Hum Retroviruses* 2003; **19**(9): 727-30.
27. Sypsa V, Psichogiou M, Paraskevis D, et al. Rapid Decline in HIV Incidence Among Persons Who Inject Drugs During a Fast-Track Combination Prevention Program After an HIV Outbreak in Athens. *J Infect Dis* 2017; **215**(10): 1496-505.
28. Bruneau J, Zang G, Jutras-Aswad D, et al. Hepatitis C spontaneous clearance may induce partial protection against reinfection among persons who inject drugs: Results from a cohort study. *Journal of Hepatology* 2015; **62**: S828.
29. Brunton C, Kemp R, Raynel P, Harte D, Baker M. Cumulative incidence of hepatitis C seroconversion in a cohort of seronegative injecting drug users. *N Z Med J* 2000; **113**(1106): 98-101.
30. Craine N, Hickman M, Parry JV, et al. Incidence of hepatitis C in drug injectors: the role of homelessness, opiate substitution treatment, equipment sharing, and community size. *Epidemiol Infect* 2009; **137**(9): 1255-65.
31. Iversen J, Wand H, Topp L, Kaldor J, Maher L. Reduction in HCV incidence among injection drug users attending needle and syringe programs in Australia: a linkage study. *Am J Public Health* 2013; **103**(8): 1436-44.
32. Sacks-Davis R, Daniel M, Roy E, et al. The role of living context in prescription opioid injection and the associated risk of hepatitis C infection. *Addiction* 2016; **111**(11): 1985-96.
33. Smyth B, O'Connor J, Barry J, Keenan E. Retrospective cohort study examining incidence of HIV and hepatitis C infection among injecting drug users in Dublin. *J Epidemiol Community Health* 2003; **57**(4): 310-1.
34. Spittal PM, Pearce ME, Chavoshi N, et al. The Cedar Project: high incidence of HCV infections in a longitudinal study of young Aboriginal people who use drugs in two Canadian cities. *BMC Public Health* 2012; **12**: 632.
35. Tsui JI, Evans JL, Lum PJ, Hahn JA, Page K. Association of opioid agonist therapy with lower incidence of hepatitis C virus infection in young adult injection drug users. *JAMA Intern Med* 2014; **174**(12): 1974-81.
36. Blome MA, Bjorkman P, Flamholc L, Jacobsson H, Molnegren V, Widell A. Minimal transmission of HIV despite persistently high transmission of hepatitis C virus in a Swedish needle exchange program. *J Viral Hepat* 2011; **18**(12): 831-9.
37. Horyniak D, Higgs P, Jenkinson R, et al. Establishing the Melbourne Injecting Drug User Cohort Study (MIX): rationale, methods, and baseline and twelve-month follow-up results. *Harm Reduct J* 2013; **10**: 11.
38. Hagan H, Pouget ER, Williams IT, et al. Attribution of hepatitis C virus seroconversion risk in young injection drug users in 5 US cities. *J Infect Dis* 2010; **201**(3): 378-85.
39. Lucidarme D, Bruandet A, Ilef D, et al. Incidence and risk factors of HCV and HIV infections in a cohort of intravenous drug users in the North and East of France. *Epidemiol Infect* 2004; **132**(4): 699-708.
40. Micallef JM, Macdonald V, Jauncey M, et al. High incidence of hepatitis C virus reinfection within a cohort of injecting drug users. *J Viral Hepat* 2007; **14**(6): 413-8.
41. Vallejo F, Barrio G, Brugal MT, et al. High hepatitis C virus prevalence and incidence in a community cohort of young heroin injectors in a context of extensive harm reduction programmes. *J Epidemiol Community Health* 2015; **69**(6): 599-603.
